# The Microalgae *Chlamydomonas* for Bioremediation and Bioproduct Production

**DOI:** 10.3390/cells13131137

**Published:** 2024-07-02

**Authors:** Carmen M. Bellido-Pedraza, Maria J. Torres, Angel Llamas

**Affiliations:** Department of Biochemistry and Molecular Biology, Campus de Rabanales and Campus Internacional de Excelencia Agroalimentario (CeiA3), University of Córdoba, Edificio Severo Ochoa, 14071 Córdoba, Spain; b22bepec@uco.es (C.M.B.-P.); bb2topom@uco.es (M.J.T.)

**Keywords:** microalga, *Chlamydomonas*, bioremediation, wastewater, high-value-added products

## Abstract

The extensive metabolic diversity of microalgae, coupled with their rapid growth rates and cost-effective production, position these organisms as highly promising resources for a wide range of biotechnological applications. These characteristics allow microalgae to address crucial needs in the agricultural, medical, and industrial sectors. Microalgae are proving to be valuable in various fields, including the remediation of diverse wastewater types, the production of biofuels and biofertilizers, and the extraction of various products from their biomass. For decades, the microalga *Chlamydomonas* has been widely used as a fundamental research model organism in various areas such as photosynthesis, respiration, sulfur and phosphorus metabolism, nitrogen metabolism, and flagella synthesis, among others. However, in recent years, the potential of *Chlamydomonas* as a biotechnological tool for bioremediation, biofertilization, biomass, and bioproducts production has been increasingly recognized. Bioremediation of wastewater using *Chlamydomonas* presents significant potential for sustainable reduction in contaminants and facilitates resource recovery and valorization of microalgal biomass, offering important economic benefits. *Chlamydomonas* has also established itself as a platform for the production of a wide variety of biotechnologically interesting products, such as different types of biofuels, and high-value-added products. The aim of this review is to achieve a comprehensive understanding of the potential of *Chlamydomonas* in these aspects, and to explore their interrelationship, which would offer significant environmental and biotechnological advantages.

## 1. Introduction: Why Microalgae and Why *Chlamydomonas*?

Microalgae represent a broad array of single-celled, photosynthetic organisms that serve as key contributors to primary production across our planet [1]. Microalgae can adopt photoautotrophic, heterotrophic, or mixotrophic modes of life, displaying a spectrum of cell sizes, shapes, and structures. Responsible for a significant portion of the global carbon capture, microalgae play a crucial role in supporting ecosystems [2]. Microalgae share a common evolutionary origin that can be traced back to a primary endosymbiotic event involving a cyanobacterium, which eventually evolved into the plastid [3]. This process has resulted in the emergence of a wide range of colorful and metabolically diverse algal groups, such as diatoms and dinoflagellates [4]. 

Microalgae are employed in activities such as wastewater treatment [5], biofuel generation [6], animal feed production [7], and the extraction of high-value-added products [8], among other applications. Additionally, microalgae show great potential as organisms for enhancing biological carbon sequestration aimed at mitigating global warming [9]. Recently, significant technical advancements, new applications, and products in microalgal biotechnology have been highlighted, showcasing how microalgae can provide high-tech, low-cost, and eco-friendly solutions for current and future societal needs [10]. This study also explores how emerging technologies, such as synthetic biology, high-throughput phenomics, and automation, can enhance the understanding of algal biology and drive the development of an algal-based bioeconomy. Consequently, microalgae hold significant ecological and economic potential.

*Chlamydomonas* is a microalga that is commonly found in freshwater and saltwater habitats, as well as in soil and snow. Taxonomically, the genus *Chlamydomonas* comprises more than 500 species [11]. Over time, it has evolved into a highly influential model organism, thanks to its numerous interesting characteristics [12]. Among the *Chlamydomonas* species, *Chlamydomonas reinhardtii* is the most commonly used due to its interesting characteristics. Among these features, *C. reinhardtii* has two flagella, grows well in axenic cultures, exhibits a relatively rapid doubling time of approximately 8–12 h, and its nuclear, chloroplast, and mitochondrial genomes are sequenced. Additionally, *C. reinhardtii* exhibited an exceptional ability to adapt and thrive under nearly all experimental conditions tested in heterotrophic, phototrophic, and mixotrophic cultivations [13]. Moreover, the *Chlamydomonas* Sourcebook [14] provides a thorough overview of essential research areas, historical background, physiology, and methodologies related to *Chlamydomonas*. Additionally, the *Chlamydomonas* Resource Centre offers a wide range of resources, including biochemical assays, protocols, plasmids, and a diverse collection of mapped mutant strains. *Chlamydomonas* biotechnology has centered on finding high-yielding strains through exploration of natural sources and improving productivity through forward genetics. Recent progress has been made in high-throughput screening, genome-wide mutant libraries, and genome editing techniques with *Chlamydomonas* [15]. Furthermore, enhancing the yield of many biotechnological processes involving *Chlamydomonas* can be achieved through synergistic interactions with other microorganisms, predominantly bacteria [16,17].

However, there are still numerous challenges hindering the efficient utilization of *Chlamydomonas* biotechnologically in bioremediation and bioproduct production. Consequently, substantial efforts are being directed towards gaining a deeper understanding of the biological mechanisms relevant to its applications. To the best of our knowledge, there has never been a single comprehensive review covering all these aspects of *Chlamydomonas*. Therefore, here we summarize and categorize these reports with the aim of highlighting the potential of *Chlamydomonas* to fulfill these tasks.

## 2. Wastewater and Advantages of Using Microalgae for Its Bioremediation

Wastewater comprises a diverse mixture of organic and inorganic compounds, as well as synthetic substances that reflect societal lifestyles and technology. Carbohydrates, fats, sugars, and amino acids are among the primary contaminants found in wastewater. Indeed, amino acids constitute three-quarters of the organic carbon in some wastewater [18]. Inorganic constituents found in wastewater include a variety of substances such as calcium, sodium, magnesium, potassium, sulfur, arsenic, bicarbonate, heavy metals, nitrates, chlorides, phosphates, and non-metallic salts [19]. Persistent organic pollutants include chlorinated and aromatic compounds, such as polychlorinated biphenyls, polycyclic aromatic hydrocarbons, and organochlorine pesticides [20]. The composition of wastewater varies depending on its source. Municipal wastewater is generated from households, commercial establishments, and institutions. It typically contains organic matter, nutrients, pathogens, and various chemicals from soaps and detergents [21]. Agricultural wastewater originates from farming activities and can contain organic matter, pesticides, herbicides, and fertilizers [22]. Industrial wastewater may include a diverse array of industry-specific pollutants, including heavy metals, organic chemicals, and oils [23]. Each type of wastewater has its own unique characteristics and requires specific treatment approaches to address its particular contaminants. 

**Figure 1:**
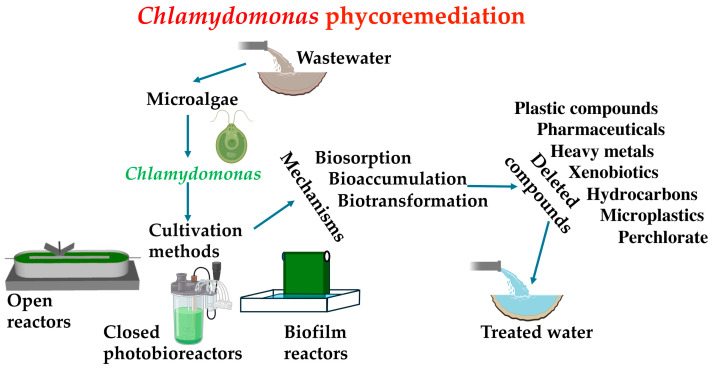
The *Chlamydomonas*-based phycoremediation process for wastewater treatment. The utilization of *Chlamydomonas* in wastewater phycoremediation is shown schematically, detailing the main cultivation methods, the employed mechanisms, and the various compounds that can be bioremediated.

As anthropogenic activities increase, resulting in more complex wastewater compositions, it becomes crucial to develop wastewater treatment procedures that are easy to implement, efficient, and environmentally friendly. Traditional methods for treating wastewater include physical, mechanical, chemical, and biological approaches (Figure 1). Physical methods entail processes such as sedimentation, screening, and skimming, while mechanical methods include filtration techniques like ceramic membrane and sand filter technology [24]. Chemical methods involve processes such as neutralization, adsorption, precipitation, disinfection, and ion exchange [25]. However, purely physical–chemical methods have proven ineffective in treating wastewater with complex compositions. Biological methods for wastewater treatment involve the use of microorganisms that consume pollutants in the wastewater as food [26]. However, biological wastewater treatment also has various drawbacks, including high energy consumption, expenses associated with aeration, and challenges in sludge management. Therefore, the integration of physical–chemical and biological methods is an effective approach for sustainable wastewater treatment [27]. 

Phycoremediation (where ‘phyco’ means algae in Greek) is a sustainable and environmentally friendly approach that utilizes various types of algae, including cyanobacteria, microalgae, and macroalgae, to remove or extract pollutants from wastewater (Figure 1). Among the benefits of phycoremediation are the removal of nutrients and xenobiotic substances, the reduction in excess nutrients from effluent with high organic material, CO_2_ mitigation, the treatment of effluents with heavy metal ions, and the monitoring of potentially toxic substances using algae as biosensors [28]. Microalgae have the ability to absorb and break down contaminants through processes such as biosorption, bioaccumulation, and biotransformation [29]. Phycoremediation not only helps in the removal of pollutants but also results in the production of algal biomass, which can be utilized for various valuable products such as food, feed, fertilizers, pharmaceuticals, and biofuels [30]. A wide range of non-pathogenic algae are utilized for wastewater treatment, such as *Chlorella* sp., *Spirulina* sp., *Scenedesmus* sp., *Nostoc* sp., and *Oscillatoria* sp., [31]. In this review, we will focus on those studies that use *Chlamydomonas* in phycoremediation.

## 3. Microalgae Cultivation Methods

Microalgae cultivation methods are categorized into suspended systems (including open reactors and closed photobioreactors) and attached systems, such as biofilm reactors (Figure 1). Open reactors include lakes and natural ponds, as well as specially designed high-rate algal ponds (HRAPs) that are tanks or lagoons featuring a paddle wheel that circulates wastewater. HRAPs can be an economical and sustainable method for treating wastewater, as microalgae efficiently absorb nutrients such as phosphorus and nitrogen, as well as help remove organic and inorganic contaminants [32]. Closed photobioreactors (PBR) are enclosed systems utilized for the cultivation of microalgae and other phototrophic microorganisms. They provide excellent control over culture conditions with minimal risk of contamination. Different types of PBRs include flat panel, tubular, and stirred tank designs [33]. The cultivation of microalgae in biofilm reactors involves immobilizing the microalgae on a surface that acts as a support, forming a continuous layer. This method offers advantages such as higher concentration per unit volume of medium, reduction or absence of cells in the effluent, and ease of harvesting [34]. The extraction and dewatering of algae cells from biofilms are simplified by the ease of separating attached cells from their growth medium. In the context of technological applications, regulating the adhesion properties of *Chlamydomonas* could significantly enhance the efficiency of biofilm reactors by controlling surface colonization and biofilm formation. So far, the basic principles governing the colonization of surfaces by motile, photosynthetic microorganisms remain largely unexplored. Interestingly, *Chlamydomonas* has the ability to secrete substances such as sulphated polysaccharides that act as antibiofilm agents for certain bacteria, preventing these bacteria from attaching to the biofilm [35]. This property can be highly beneficial in controlling the occurrence of bacterial contaminations. 

## 4. *Chlamydomonas* Phycoremediation

Microalgae, particularly *Chlamydomonas*, exhibit a remarkable capacity and diversity in bioremediating various molecules. Next, we will present the main mechanisms for bioremediation. Biosorption is a passive mechanism whereby microalgae serve as a biological sorbent to capture and accumulate pollutants. Microalgae utilize their cell wall and various chemical groups to attract and retain contaminants [36]. Microalgae can remove pollutants through bioaccumulation. The main differences between biosorption and bioaccumulation processes lie in their mechanisms. Biosorption is a passive process where microorganisms utilize their cellular structure to capture pollutants on the binding sites of the cell wall. On the other hand, bioaccumulation is an active process that involves the accumulation of pollutants in the biomass of microalgae, either by accumulation or uptake into intracellular spaces [37]. Bioaccumulation requires cellular growth and is typically slower than biosorption. Biotransformation involves the breakdown of pollutants, either inside or outside the cells, facilitated by enzymes [38]. While there are not significant concerns with biosorption and bioaccumulation, biotransformation presents more challenges due to the possibility of its products being potentially more toxic than the original compounds. 

Some studies have cultivated *Chlamydomonas* in PBRs for the decontamination of wastewater [39] (Table 1). In this regard, *Chlamydomonas debaryana* using dairy wastewater reduced nitrogen, phosphorus, organic carbon, and chemical oxygen demand by more than 85% [40]. *C. debaryana* and *C. reinhardtii* were able to effectively treat swine wastewater [41]. With *C. reinhardtii*, 55.8 mg of nitrogen and 17.4 mg of phosphorus per liter per day were effectively removed from industrial wastewater [42]. Using *C. mexicana*, a high removal efficiency of nitrogen (62%), phosphorus (28%), and inorganic carbon (29%) was achieved in piggery wastewater [43]. Research shows that nitrogen-limited wastewater microalgae can be effectively used for biomass production through anaerobic fermentation [44]. Wastewater collected from a paper industry was treated using *C. reinhardtii*, resulting in significant reductions in nitrate (86%), phosphate (88%), and chemical oxygen demand (COD) (93%) [45]. 

Numerous studies have reported the use of HRAP in wastewater treatment, primarily focusing on genera such as *Scenedesmus* and *Chlorella* [46]. However, very few records exist of applying HRAP with *Chlamydomonas*. In a pilot-scale HRAP experimental wastewater treatment, *Chlamydomonas* sp. was found to be one of the dominant genera. The study reported a reduction in the biochemical oxygen demand by 90%, chemical oxygen demand by 65%, total nitrogen by 46%, and total phosphorus by 20% [47]. A study on the bioremediation of piggery wastewater using HRAP revealed that *Chlamydomonas* sp. was the dominant species, with average chemical oxygen demand and total nitrogen removal efficiencies of 76% and 88%, respectively [48]. In another study employing HRAP with *Chlamydomonas* sp. for treating municipal wastewater, average reductions in volatile suspended solids, total nitrogen, and biochemical oxygen demand were 63%, 76%, and 98%, respectively [49]. 

*Chlamydomonas* sp. JSC4 has been successfully employed in a biofilm reactor for the removal of phosphorus, nitrogen, and copper from swine wastewater [50]. In a biofilm reactor, *Chlamydomonas pulvinata* TCF-48 g has demonstrated significant polyphosphate accumulation and a high phosphorus removal rate of 70%, making it valuable for phosphate recovery applications [51]. The encapsulation of *C. reinhardtii* in alginate beads has been successfully carried out to remove various types of contaminants such as phosphorus, nitrogen, lead, mercury, and cadmium [52] or even phenol [53].

*C. reinhardtii* has shown a significant capability for biosorption, effectively removing copper, boron, manganese [54], arsenic [55], nickel [56], zinc, cadmium [57], and uranium [58]. In *C. reinhardtii*, gene manipulation has been conducted to enhance the expression of the metal tolerance proteins metallothioneins [59], resulting in increased tolerance to cadmium [60], chromium [61], copper [62], mercury [63], and lead [64]. Biosorption in *C. reinhardtii* as a defense mechanism against silver nanoparticles involves an increase in phytochelatin and exopolysaccharides content, along with a decrease in glutathione levels [65]. *C. reinhardtii* has been shown to bioaccumulate several compounds such as Prometryne (herbicide) [66], o-nitrophenol [67], and *C. mexicana* carbamazepine (antiepileptic agents) [68]. 

Some of the pollutants removed via biotransformation by *C. reinhardtii* include organophosphorus pesticide such as trichlorfon [69], polycyclic aromatic hydrocarbons such as benz(a)anthracene [70] and polystyrene [71], and microplastics such as bisphenol A [72]. The pharmaceuticals products that can be biotransformed by microalgae have been reviewed in [73]. Among these, *Chlamydomonas* has demonstrated high efficiency with the following compounds: *Chlamydomonas* sp. with 7-amino-cephalosporanic acid [74], *C. mexicana* with enrofloxacin [75], and *C. reinhardtii* with carbamazepine, ciprofloxacin, erythromycin, estrone, norfloxacin, ofloxacin, paracetamol, progesterone, roxithromycin, salicylic acid, sulfadiazine, sulfadimethoxine, sulfametoxydiazine, sulfamethazine, triclocarban, triclosan, trimethoprim [76], sulfadiazine [77], and ibuprofen [78]. *C. reinhardtii* has been found to biotransform antibiotics like azithromycin, erythromycin, and sulphapyridine [79]. *C. reinhardtii* was shown to be able to biotransform the hormones β-estradiol and 17α-ethinylestradiol [80] as well as the non-steroidal anti-inflammatory drug diclofenac [81]. 

*Chlamydomonas* can metabolize xenobiotics through a wide range of enzymatic processes, including CYP450 oxidation reactions, hydrolysis, glutamate conjugation, and methylation [82]. *Chlamydomonas moewusii* excretes laccases capable of breaking down and detoxifying phenolic pollutants [83]. The toxicity responses of different pollutants, such as benzophenone-3, bisphenol A, oxytetracycline, and atrazine, in *C. reinhardtii* showed a similar pattern: an increase in chlorophyll autofluorescence and a decrease in growth rate and vitality [84]. The biotransformation of five bisphenol derivatives (AF, B, F, S, and Z) by *C. mexicana* shows that all the biotransformed products were less toxic than the parent compounds [85]. *Chlamydomonas* has also been used in efforts to degrade commonly used plastic components such as Polyethylene terephthalate (PET). In *Ideonella sakaiensis*, a novel plastic degradation enzyme called PETase has been identified [86]. The *I. sakaiensis* PETase has been expressed through genetic recombination in the *C. reinhardtii* nucleus and chloroplast genomes, showing a significant ability to break down PET [87]. Under specific adverse conditions, such as NaCl stress, EDTA exposure, or acidic pH, *C. reinhardtii* can form multicellular aggregates called palmelloids. These are small clonal structures that result from cells failing to separate after division [88]. The defense mechanisms of *C. reinhardtii* under perchlorate stress were investigated, revealing palmelloid formation when exposed to 100 and 200 mM perchlorate [89]. These researchers highlight the metabolic versatility of *Chlamydomonas* in dealing with xenobiotic compounds, demonstrating its ability to transform and process a variety of chemicals through different mechanisms. The encapsulation of microalgae is a process in which the microalgae are coated with a protective layer to enhance their stability, protect them from adverse conditions, and facilitate their application. This process offers various biotechnological advantages, such as protecting the formation of bioactive compounds, promoting release control, improving solubility, and enhancing bioavailability [90]. Various materials, including alginate, carrageenan, chitosan, and polyvinyl, have been used for the immobilization of microalgae [91]. In the case of *Chlamydomonas*, alginate has been the most successful and currently the most commonly used material for encapsulation. The pore size of alginate beads in *C. reinhardtii* is critical, with the highest efficiency for contaminant removal obtained in gel beads with a pore size of 3.5 mm [92]. In this regard, *Chlamydomonas* cells immobilized with carboxymethyl cellulose beads have demonstrated a great capacity for decontaminating Uranium (VI) through biosorption [93]. 

Microalgae have been actively employed in initiatives focused on reducing CO_2_ emissions due to their ability to absorb CO_2_ via photosynthesis. *C. reinhardtii* exhibits a superior ability to fix CO_2_ compared to other photosynthetic organisms [94]. Bio-fixation refers to the process by which certain organisms, such as microalgae, utilize CO_2_ from the air or other sources like flue gas streams to create biomass. The production of 1 g of microalgae biomass leads to the sequestration of 1.8 g of CO_2_ [95]. In *Chlamydomonas* the expression of a single H^+^-pump increase its tolerance to high concentrations of CO_2_, such as those found in industrial flue gas [96]. These findings illustrate the potential of *C. reinhardtii* to mitigate CO_2_ emissions from industrial sources. 

The studies mentioned regarding bioremediation with *Chlamydomonas* present several limitations that we will now outline, which we believe could be addressed in future research. Many studies are conducted at the laboratory or pilot scale. For practical application, it is crucial to evaluate the effectiveness of *Chlamydomonas* under real conditions, such as in large-scale wastewater treatment plants. Studies often focus on a single species or strain of *Chlamydomonas*. Investigating a broader range of species and strains would be beneficial to better understand their bioremediation potential. The interaction of *Chlamydomonas* with other microbial species in the natural environment can impact its efficacy. Studying these interactions and their impact on bioremediation is essential. Some studies mention the biotransformation of contaminants, but the toxicity of the resulting products is not always evaluated. Investigating the effects of these transformed products is critical. Cultivation parameters (such as light, temperature, pH, and nutrient concentration) can affect the efficiency of *Chlamydomonas*. Optimizing these conditions could improve outcomes. Many studies are short-term. Researching the long-term stability and efficacy of *Chlamydomonas* in wastewater treatment systems is essential. In summary, improving these studies requires a combination of more realistic experimental approaches, species diversity, toxicity evaluation, and cultivation-condition optimization. These efforts will contribute to a more effective application of *Chlamydomonas* in bioremediation.

## 5. *Chlamydomonas* Bioproduct Generation

### 5.1. Biomass

One of the main products derived from the cultivation of microalgae is their biomass, as it is used as raw material for obtaining other derived bioproducts. It would be highly beneficial economically to use the biomass resulting from the bioremediation process for bioproduct purification. However, the utilization of biomass derived from wastewater treatment encounters several inherent challenges. These challenges include the scalability of biomass production, the presence of xenobiotics and heavy metals, as well as the contamination with bacteria, fungi, and viruses, all of which limit their extensive application [97]. Although numerous efforts are being made to address this issue, the production of the main bioproducts obtained from microalgae still does not use wastewater as a cultivation source. Next, we will present studies that utilize *Chlamydomonas* to obtain certain bioproducts, some of which use biomass derived from wastewater remediation.

**Figure 2:**
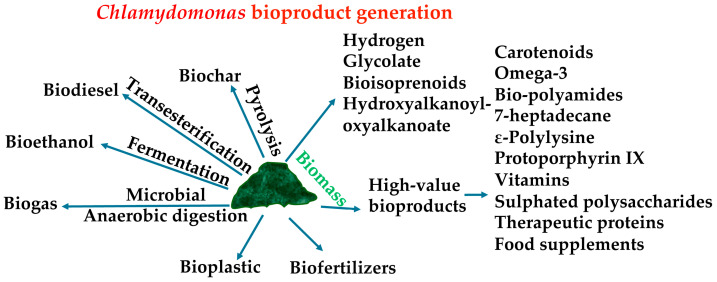
The process of *Chlamydomonas* bioproduct generation. *Chlamydomonas* cells can be cultivated in various conditions. After cultivation, the collected biomass and medium can then be processed as indicated to obtain the specified bioproducts (see Table 2).

The composition of biomass is influenced by the strains of microalgae and the culture conditions [98]. One straightforward method to increase biomass productivity involves altering the culture medium conditions or adjusting the supply of certain macroelements. For example, in microalgae, some researchers have evaluated the effect of various carbon sources [99], pH variations [100], and photoperiods [101], as well as trace element compositions for biomass production [102]. Consequently, various approaches have been explored to optimize microalgae biomass enriched in specific biomolecules (Figure 2). The highest biomass concentration of *Chlamydomonas* obtained so far has been heterotrophically with acetate, reaching 23 g/L [103] (Table 2), far behind other green algae that are able to consume glucose as a substrate, like *Chlorella* sp. and *Scenedesmus* sp., for which biomass reached 271 g/L and 286 g/L, respectively [104].

### 5.2. Biochar

Biochar is a carbonaceous material produced through the pyrolysis of biomass (Figure 2), which can be obtained from microalgae, agricultural residues, wood, or organic waste [105]. Biochar is characterized by its high porosity and specific surface area, making it useful for improving soil quality and carbon sequestration. It is used in agriculture as a soil amendment to enhance soil structure, retain nutrients and water, and promote beneficial microbial activity. Additionally, biochar is considered a strategy for mitigating climate change, as burying it in the soil can store carbon stably for long periods [106]. *C. reinhardtii* biomass has been successfully used to prepare biochar [107]. The highest biochar yield was 93.9%, achieved through dry torrefaction at 200 °C using *Chlamydomonas* sp. JSC4 [108]. Biochar prepared from *Chlamydomonas* sp. has been shown to have a high capacity for removing contaminants [109] (Table 2).

### 5.3. Biofertilizers

Microalgae are used as a biofertilizers and biostimulants by promoting crop growth and increasing soil nutrient contents, thereby reducing the usage of chemical fertilizers [110]. In contrast, *Chlamydomonas* species have received little attention and are not fully utilized in agriculture, despite being among the most abundant microalgae species in natural soil ecosystems. In this regard, a study on the effects of *Chlamydomonas applanata* M9V as a biofertilizer on wheat found that it performed even better than a certain amount of chemical fertilizer [111]. Acid-hydrolyzed dry biomass of *C. reinhardtii* improved the phosphorus, nitrogen, and carotenoid contents of *Solanum lycopersicum* [112]. The application of live *Chlamydomonas* cells significantly increased leaf size, shoot length, fresh weight, number of flowers, and pigment content of *Medicago truncatula* [113]. Lyophilized powders derived from *C. reinhardtii* have been found to positively affect the growth of maize plants by producing bioactive compounds that act as biostimulants, enhancing plant growth, crop performance, yields, and quality [114]. Biomass extracts of *Chlamydomonas* sp. exhibited auxin-like activity that increased the number of roots in cucumber plants [115]. *Chlamydomonas sajao* can improve soil physical properties, such as aggregation and stability, thereby contributing to enhanced soil structure and nutrient retention [116]. These results suggest that *Chlamydomonas* can be an effective alternative to chemical fertilizers for promoting crop growth and yield (Table 2).

### 5.4. Bioplastic

Bioplastics are biodegradable materials derived from renewable biomass sources, offering a sustainable alternative to traditional plastics [117]. Various molecules can be used as building blocks for bioplastics, including polyhydroxybutyrate (PHB), starch, TAG, lactic acid, or polybutylene succinate. PHB can be naturally synthesized by certain bacteria, such as *Azotobacter* or *Pseudomonas*. PHB production involves three key enzymes: β-ketothiolase, acetoacetyl-CoA reductase, and PHB synthase, encoded by *phbA*, *phbB*, and *phbC*, respectively. Research has focused on engineering *Chlamydomonas* strains to enhance PHB de novo biosynthesis, as *Chlamydomonas* naturally cannot synthesize PHB. With this aim, the *phbB* and *phbC* genes from *Ralstonia eutropha* have been inserted into the *C. reinhardtii* genome, leading to the observation of PHB granules in the cytoplasm [118] (Table 2). While cytosolic accumulation of PHB in *Chlamydomonas* often results in impaired cell growth and low yield, peroxisomes have emerged as a promising alternative. A complete PHB biosynthesis pathway has been successfully reconstructed by expressing the three PHB synthesis genes and targeting the proteins to the peroxisomes. Within the peroxisomes of these strains, PHB reached 21.6 mg/g, which represents a 3600-fold increase over cytosolic PHB production [119]. Another strategy is to use TAG as the building block for bioplastics. TAG synthesized by *C. reinhardtii* has been directly crosslinked with glycerol or ammonium persulfate and molded into plastic beads that are capable of withstanding compressive stress up to 1.7 megapascals [120]. Cell-plastics are a type of bioplastic that directly utilizes raw cells and the hydrolyzed cell broth. Unlike conventional bioplastics, cell-plastics do not require exhaustive processes for extracting and refining the biomolecules that serve as the building blocks. Recently, *Chlamydomonas* cells have arisen as the constituent blocks of this new type of bioplastic, as their cell size and protein-rich, cellulose-free cell wall were demonstrated to be ideal components for its fabrication [121]. 

### 5.5. Biofuels

Biofuels are fuels derived from renewable biological sources such as plants or plant-derived materials. First-generation biofuels are produced from food crops. Second-generation biofuels are derived from non-food sources such as waste, and third-generation biofuels are produced from sources that do not compete with arable land, such as microalgae [122]. Microalgae have regained attention as alternative resources for environmentally friendly production of biofuels, including biodiesel, bioethanol, biogas, and biohydrogen. These biofuels can be produced through thermochemical and biochemical conversions, photosynthesis-mediated microbial fuel production, and transesterification [123]. 

#### 5.5.1. Biodiesel

Triacylglycerols (TAG) are crucial lipids in microalgae for biofuel production. Oleaginous microalgae, rich in TAG, can be converted into biodiesel through transesterification, a process that transforms TAG into fatty acid methyl esters, the key components of biodiesel [124]. Utilizing *Chlamydomonas* sp. JSC4, a direct transesterification process was employed, resulting in nearly 100% biodiesel production in a single step [125] (Table 2). Given that biodiesel production is closely linked to the quantity of lipids and TAGs, various strategies have been explored to enhance their production in *Chlamydomonas*. Some studies have focused on elucidating the functions of key genes involved in lipid and TAG production. The down-regulation of the phosphoenolpyruvate carboxylase gene in *C. reinhardtii* resulted in a 74.4% increase in lipid content [126]. The overexpression of acetyl-CoA synthetase resulted in a 2.4-fold increase in the accumulation of TAG [127]. In *C. reinhardtii*, the mutation of *ACX2*, which encodes a member of the acyl-CoA oxidase responsible for the first step of peroxisomal fatty acid beta-oxidation, resulted in an accumulation of 20% more lipid [128]. A mutant of *C. reinhardtii* deficient in phospholipase showed an increase in TAG content of up to 190% [129]. The overexpression of the ferredoxin gene *PETF* in *C. reinhardtii* resulted in higher lipid content [130]. The Target of Rapamycin (TOR) plays a crucial role in regulating cell growth. It has been shown that mutants of *C. reinhardtii* lacking TOR experience an increase in TAG production [131]. The strategy of heterologously overexpressing genes in *Chlamydomonas* has been successful in increasing TAG content. In this sense, the heterologous expression of the *Dunaliella tertiolecta* fatty acyl-ACP thioesterase in *C. reinhardtii* leads to increased lipid production [132]. By expressing the diacylglycerol acyltransferase from *Saccharomyces cerevisiae* into *C. reinhardtii*, the fatty acids and TAG content increased by 22% and 32%, respectively [133]. The heterologous expression of *Lobosphaera incisa* glycerol-3-phosphate acyltransferase in *C. reinhardtii* enhances TAG production [134]. The synthesis of starch and lipids competes for carbon skeletons; thus, inhibiting starch synthesis is another strategy for increasing TAG production. In this sense, silencing ADP-glucose pyrophosphorylase in *C. reinhardtii* resulted in a tenfold increase in TAG content [135]. Genetically modifying *Chlamydomonas* sp. JSC4 in the gene that encodes the starch debranching enzyme promotes carbohydrate degradation and redirects carbon resources into lipids, resulting in a 1.46-fold increase in lipid content [136]. 

A commonly employed approach to accumulate TAG in *Chlamydomonas* is to induce stress conditions, particularly nutrient limitation or starvation [137]. *C. reinhardtii* exhibits a notable increase in TAG accumulation under low nitrogen concentrations [138]. Under nitrogen deprivation, *C. reinhardtii* starch mutants exhibit almost a 10-fold increase in TAG [139]. Under nitrogen limitations, increasing the expression of S-adenosylmethionine synthetase in *C. reinhardtii* enhances cell viability and TAG production [140]. Phosphorus stress also triggers TAG production in *Chlamydomonas* [141]. Additionally, a higher TAG content is generated under conditions of low sulfur concentration [142]. The lipid content in *C. mexicana* was observed to rise as the concentration of NaCl was increased to 25 mM [143]. The lipid content of the *C. reinhardtii* starchless mutant BAF-J5 increased by 76% following a temperature shift to 32 °C [144]. 

Increasing TAG levels by inducing stress conditions often comes at the expense of inhibited microalgal growth. Under these conditions, there is an inverse relationship between TAG yield and microalgal growth. To mitigate this, it has been reported that overexpressing the transcription factor MYB1 in *C. reinhardtii*, which mediates lipid accumulation, results in nearly 60% more TAG without negatively impacting cell growth [145]. In another strategy, a cultivation approach involving two stages has been proposed, wherein *C. reinhardtii* experiences nutrient stress only after an initial period of optimal growth, allowing for high TAG accumulation [146]. The development of effective methods for cultivating *Chlamydomonas* is essential in biodiesel production. In this regard, in *C. reinhardtii*, a multi-parametric kinetic model developed using computational tools has been proven, resulting in significant increases in lipids (74%) [147].

#### 5.5.2. Bioethanol

Bioethanol is a biofuel that can be obtained through the fermentation of various types of biomass containing high amounts of sugars. For bioethanol production, the high carbohydrate content present in both the cellulose and hemicellulose cell walls, as well as the starch-based cytoplasm, is broken down into monomeric sugars during enzymatic hydrolysis prior to fermentation. However, the cell wall of *Chlamydomonas* is not made of cellulose like in plants, but of five dense, glycoprotein-rich layers [148]. Therefore, efforts have been focused on utilizing starch-rich *Chlamydomonas* for the production of bioethanol. The biomass of *C. reinhardtii* UTEX 90 was converted into glucose through two hydrolytic steps using α-amylase and amyloglucosidase, with nearly all the starch successfully transformed into glucose without damaging the cell wall, reducing the costs of bioethanol purification [149] (Table 2). Pretreating *C. reinhardtii* UTEX 90 biomass with sulfuric acid (1–5%) at temperatures ranging from 100 to 120 °C significantly increases the glucose release for the production of bioethanol [150]. The supraoptimal temperature treatment method, which involved cultivating *C. reinhardtii* at 39 °C despite its optimal temperature being 25 °C, was successfully applied and resulted in nearly a threefold enhancement of starch content [151]. The hormones have also been described to have a very important role in starch accumulation; in this sense, in *Chlamydomonas*, 100 µM of Indole-3-acetic acid produces an accumulation of up to nine-times more starch [152]. *Chlamydomonas* sp. QWY37 has been effectively utilized for bioethanol production from swine wastewater, achieving a maximum bioethanol yield of 61 g/L [153]. 

#### 5.5.3. Biogas

Biogas is a renewable energy source primarily composed of CH_4_, derived from the microbial anaerobic digestion of biomass obtained from various sources (Figure 2). The production of biogas involves multiple stages, including hydrolysis, acidogenesis, acetogenesis, and methanogenesis, which are facilitated by a microbial consortium that plays a crucial role in influencing both the composition and yield of the biogas [154]. This process eliminates the need to extract specific macromolecules, such as lipids, proteins, or carbohydrates, and can be carried out using wet biomass [155]. The fermentation of *C. reinhardtii* biomass produces approximately 587 mL of biogas per gram of volatile solids [156]. However, microalgae biomass is not ideal for biogas generation due to its high protein content, which results in an unfavorably low carbon-to-nitrogen ratio. This imbalance arises because the ammonia released during protein degradation inhibits the methanogenesis process [157]. *C. reinhardtii* biomass has been studied for its potential in overcoming this limitation. In this regard, the anaerobic digestion of *C. reinhardtii* biomass obtained in low-nitrogen media has shown remarkable efficiency in biogas production due to its high carbon-to-nitrogen ratio [158] (Table 2). 

The high resistance of microalgae biomass to microbial decomposition due to their rigid cell walls is a significant challenge in biogas production. However, since the main components of the *C. reinhardtii* cell wall are glycoproteins rather than cellulose, *C. reinhardtii* has been shown to produce larger quantities of biogas compared to species with more complex cell walls (such as *Chlorella* sp. and *Scenedesmus* sp.) [159]. The findings revealed that the *C. reinhardtii* cell wall was not an obstacle but instead became advantageous by enabling the gradual degradation of intracellular content [160]. One way to valorize the microalgal biomass produced during wastewater treatment is to utilize it as a source for biogas production, thereby reducing the economic costs of treatment [161]. In this regard, *Chlamydomonas* sp. *Ck* has demonstrated high efficiency in decontaminating piggery wastewater while simultaneously producing a high biogas yield [44]. For all the reasons mentioned, anaerobic digestion of *C. reinhardtii* biomass can be considered a cost-effective alternative for biogas production compared to other methods.

#### 5.5.4. Hydrogen

The production of the preceding bioproducts shares the common step of first obtaining biomass, and then extracting these compounds from it. Next, we will present some products that *Chlamydomonas* releases into the culture medium and therefore can be purified without needing to be extracted from the biomass, thereby reducing the economic cost of their production (Figure 2). A prominent example of this is hydrogen, which has emerged as one of the most promising energy carriers for future energy demands. Hydrogen presents the opportunity to cultivate living organisms such as bacteria, cyanobacteria, and microalgae capable of releasing H_2_ into the media [162]. Hydrogen is generated through enzymes known as hydrogenases [163]. *Chlamydomonas* has two hydrogenases that have been extensively studied with the aim of increasing their production efficiency [164]. The hydrogenases catalyze the reduction of protons into H_2_ either using energy from light (biophotolysis) or by oxidizing organic compounds such as starch (dark fermentation). One of the primary biotechnological challenges of using *Chlamydomonas* as a factory to produce H_2_ is the rapid inactivation of its hydrogenases by oxygen, particularly considering that oxygen is generated during photosynthesis. Therefore, the initial evidence indicating that *Chlamydomonas* was capable of producing H_2_ was observed with *Chlamydomonas moewusii* under anaerobic condition [165], and subsequently with *C. reinhardtii,* also anaerobically [166]. The first successful strategy demonstrating significant and consistent H_2_ production under aerobic conditions involved using sulfur-starved *C. reinhardtii* [167]. The reason for this is that the absence of sulfur blocks protein synthesis, thereby halting photosynthesis and oxygen production. Alternative strategies for H_2_ production under non-stress conditions are also possible, particularly in media containing acetate, which is compatible with *Chlamydomonas* growth [168,169]. However, the rates of H_2_ production under non-stress conditions are lower compared to those under stressful conditions [170]. 

In *Chlamydomonas*, numerous genetically engineered strains have been developed to enhance H_2_ production. One of the most successful approaches has been to improve the intrinsic oxygen tolerance of hydrogenase through mutagenesis [171]. A production of 1200 mL of H_2_ per liter has been reported after 6 days using the Photosystem I (PSI) cyclic electron transport mutant *pgr5*, which is defective in thylakoid proton gradient regulation [172]. Another strategy is diverting electron flow to the hydrogenase [173], and degrading or inhibiting the function of Photosystem II (PSII) to prevent oxygen production [174] (Table 2). However, strategies that do not degrade PSII appear to be advantageous, as the long-term loss of PSII inhibits cell growth. In this sense, a PSI-hydrogenase chimera was created by inserting the HydA sequence into the PsaC (stromal subunit of PSI). This redirects photosynthetic electron flow towards proton reduction [175]. A disadvantage in the use of *Chlamydomonas* is that the hydrogen production rate is influenced by the size of microalgae cells. The hydrogen production rate of *Chlorella* is higher than that of *Chlamydomonas* due to its relatively smaller size [176].

### 5.6. High-Value Bioproducts

The term “high-value bioproducts” refers to a wide range of products derived from various sources, which economically have a higher value compared to low- to medium-value products. *C. reinhardtii* is a promising organism for the production of high-value bioproducts [177]. Glycolate, a high-value cosmetic ingredient, can be overproduced in *Chlamydomonas*. When *Chlamydomonas* is in an environment with low CO_2_ (0.04%), rubisco oxygenates ribulose-1,5-bisphosphate instead of carboxylating it, consequently producing glycolate. In *Chlamydomonas*, glycolate is toxic, prompting an active system to excrete it. To facilitate the recovery of potentially lost carbon, the genes for photorespiratory metabolism are induced. Photorespiration detoxifies and recycles glycolate, generating glycerate and releasing CO_2_. In *Chlamydomonas*, glycolate dehydrogenase (GDH) is involved in photorespiration by oxidizing glycolate to glyoxylate. It has been observed that *Chlamydomonas GDH* mutants over-accumulate glycolate in the media [178]. *Chlamydomonas* has a CO_2_-concentrating mechanism (CCM) to prevent the rubisco oxygenation reaction and, consequently, glycolate excretion [179]. CIA5 is the primary transcription factor that induces the CCM, and its mutation has been shown to increase the amount of excreted glycolate [180]. By incorporating 6-Ethoxy-2-benzothiazolesulfonamide (EZA), a CCM inhibitor, glycolate production can be maximized without compromising cell viability. Under these conditions, glycolate accumulates in the medium, reaching a concentration of up to 41 mM [181]. In photorespiration, hydroxypyruvate is converted to glycerate by hydroxypyruvate reductase (HPR). In *C. reinhardtii*, the mutation of *hpr1* results in increased excretion of glycolate into the medium [182] (Table 2).

Bioisoprenoids are natural compounds synthesized by plants, animals, and microorganisms through the isoprenoid biosynthetic pathway. These compounds are structurally and functionally diverse, with a wide range of applications, including their use as perfumes, cosmetics, pigments, medicines, and chemical signals. Bioisoprene production has gained attention due to its sustainability and efficiency compared to petrochemical sources [183]. It has been demonstrated that *C. reinhardtii* can be genetically modified to produce significant amounts of bioisoprene by overexpressing four different plant isoprene synthase genes (*IspS*), with the strain expressing the *Ipomoea batatas IspS* gene showing the highest isoprene levels [184] (Table 2). 

Hydroxyalkanoyloxyalkanoates (HAA) are a type of lipidic surfactants that can be produced by certain bacteria that show great potential for a wide range of applications. They are synthesized by the condensation of hydroxyalkanoic acids, which are produced by the metabolism of fatty acids. The chloroplast genome of *C. reinhardtii* was engineered by inserting the gene encoding the acyltransferase of *P. aeruginosa*, a key enzyme in HAA synthesis, resulting in high concentrations of HAA not only in the intracellular fraction but also in the extracellular [185].

There is strong interest in developing bio-based hydrocarbons and their unsaturated analogs, the alkenes, as potential substitutes for hydrocarbons derived from petroleum. The alkene 7-heptadecene has high demand for various biotechnological processes. While the biological function of alkenes in microalgae remains completely unknown, it has been shown that in *C. reinhardtii*, the enzyme fatty acid photodecarboxylase is responsible for synthesizing 7-heptadecene [186] (Table 2). This discovery opens the possibility of overproducing this alkene in *C. reinhardtii*. ε-Polylysine is a biodegradable polymer composed of 25–30 lysine monomers that has a variety of applications, including antimicrobial activity and anticancer agent [187]. It has been reported that ε-polylysine is produced from *Chlamydomonas* sp. supplemented with lysine, aspartate, and tricarboxylic acids, achieving a maximum production of 2.24 g/L [188].

Bio-polyamides, also known as nylons, are sustainable polymers derived from renewable resources. Bio-polyamides have excellent material properties, leading to a high demand for polyamide plastics with diverse applications across various industries [189]. Cadaverine and putrescine are polyamines commonly used as precursors and building blocks for the synthesis of bio-polyamides. By the heterologous expression of two *E. coli* lysine decarboxylases in *C. reinhardtii*, it was possible to significantly enhance the synthesis of cadaverine [190]. The mutation of essential genes in the *C. reinhardtii* polyamine biosynthesis pathway identified ornithine decarboxylase 1 (ODC1) as a crucial regulator that controls the accumulation of putrescine. Subsequently, the authors overexpressed different ODCs, resulting in a significant increase in cellular putrescine levels, reaching a maximum yield of 200 mg/L [191] (Table 2). This achievement marks the first instance of microalgal bio-production of putrescine.

*C. reinhardtii*, *Chlorella vulgaris*, *Dunaliella bardawil*, *Arthrospira platensis*, *Auxenochlorella protothecoides*, and *Euglena gracilis* are among the very few microalgae recognized by the Food and Drug Administration as Generally Recognized as Safe (GRAS) organisms [177]. This acknowledgment allows their use as a nutritional component in food, presenting new opportunities for the utilization of *C. reinhardtii*. Clinical studies on the human consumption of *C. reinhardtii* whole cells have demonstrated positive effects on gastrointestinal health and microbiota, showing that the intake of C. reinhardtii cells promotes microbiota eubiosis, reducing imbalances and improving the overall health of the intestine [192]. The development of alternative plant-based products to substitute meat has led to the exploration of heme-containing proteins for their ability to provide a meat-like color and flavor. One such compound that can provide these qualities is protoporphyrin IX (PPIX) a crucial intermediate in the heme biosynthetic pathway. In this regard, engineered C. reinhardtii strains have been shown to overexpress PPIX [193]. 

Antioxidants are widely recognized for their beneficial impact on health and their crucial role in protecting cells from the harmful effects of free radicals. *Chlamydomonas agloeformis* has garnered attention due to its exceptionally high antioxidant capacities that surpass those of higher plants [194]. Carotenoids are a diverse group of lipid-soluble pigments produced by plants and microorganisms, known for their benefits as vitamin precursors and antioxidants. Astaxanthin, a ketocarotenoid, is recognized as one of the most powerful natural antioxidants among carotenoids [195]. Astaxanthin is currently primarily produced industrially from the microalgae *Haematococcus pluvialis*, with the crucial enzyme involved in its biosynthesis being β-carotene ketolase (BKT) [196]. The synthetic redesign and overexpression of *C. reinhardtii BKT* has been shown to achieve Astaxanthin productivities of up to 4.3 mg/L/day, which is comparable to the results obtained with *H. pluvialis* [197] (Table 2). This production does not impair the growth or biomass productivity of *C. reinhardtii*, presenting a promising alternative to natural astaxanthin-producing algal strains. Furthermore, the accumulation of astaxanthin has led to enhanced high-light tolerance and increased biomass productivity [198]. Blocking the expression of *ATG1* and *ATG8*, genes involved in autophagy in *C. reinhardtii*, leads to a 2.3-times increase in carotenoid biosynthesis, indicating that autophagy does play a role in regulating carotenoid levels [199].

*Chlamydomonas* has been shown to be able to synthesize vitamins C, A, E, B_1_, B_7_, B_9_, and ergosterol, the precursor of vitamin D_2_ [200]. However, for most of these vitamins, the mechanisms regulating their synthesis to achieve overproduction have not been studied in detail. In *C. reinhardtii*, oxidative stress leads to a substantial increase in vitamin C levels [201]. Omega-3 fatty acids play critical roles as nutrients and are extensively utilized in medicine. A comparison of *C. reinhardtii* with *Chlorella* and *Spirulina* revealed that *C. reinhardtii* contains superior amounts of omega-3 fatty acids, both in quality and quantity [202]. Sulphated polysaccharides (SPs) are polymer chains containing one or more monosaccharide units that have been modified with sulfate groups. *C. reinhardtii* is capable of synthesizing SPs, which have been associated with several beneficial properties, including potent antioxidant and anticancer effects [203], antineurodegenerative effects [204], and antibiotic effects [205].

More than 40 therapeutic proteins, such as antibodies, enzymes, viral proteins, and hormones, among others, have been successfully expressed in *C. reinhardtii* [206]. ICAM-1, a protein belonging to the immunoglobulin superfamily, was targeted for secretion into the extracellular media and was found to be fully active, suggesting that *C. reinhardtii* can produce mammalian proteins that are correctly folded and functional. Additionally, it achieved a concentration of up to 46.6 mg/L, marking the highest reported concentration of any recombinant protein in *C. reinhardtii* to date [207] (Table 2). The production of full-length spike protein, a crucial component for the infectivity of SARS-CoV-2, has been successfully achieved in *C. reinhardtii* as a secreted protein [208]. This achievement is crucial as it offers a simpler and more economical platform for producing recombinant spike proteins in microalgae.

**Table 2:** Table summarizing the main characteristics of the different bioproducts generated by *Chlamydomonas*.

Microalgae	Bioproduct	Experimental Condition	Productivity/Characteristic	References
*Chlamydomonas reinhardtii* CC-2937	Biomass	Erlenmeyer flasks containing 50 mL of Tris-acetate-phosphate media on a shaker under constant light of 75 µmol photons m^−2^ s^−1^	23 g/L	[103]
*Chlamydomonas* sp.	Biochar	Bioreactor, Tris-acetate-phosphate with nitrate at 28 °C, light intensity of 150 µmol photons m^−2^ s^−1^, and bubbled with 3% CO_2_	94% *w*/*w* dry biomass	[107]
*Chlamydomonas* sp. JSC4	Biochar	Bioreactor, Tris–acetate-phosphate at 25 °C, light intensity of 70 µmol photons m^−2^ s^−1^, and bubbled air-CO_2_ (*v*/*v*, 97/3)	93.9% *w*/*w* dry biomass	[108]
*Chlamydomonas* sp. Tai-03	Biochar	Photoautotrophic mode using BG-11 medium at 26 °C, continuous aeration of 2.5% CO_2_, and light intensity of µmol photons m^−2^ s^−1^	95.4% *w*/*w* dry biomass	[109]
*Chlamydomonas applanata* M9V	Biofertilizer	Allen Arnon medium with Imipenem at 100 µg mL^−1^ and incubated for a week at 25.5 °C after shaking at 200 rpm for 24 h	Increased soil organic matter by 1.77–23.10%, total carbon by 7.14–14.46%, and C:N ratio by 2.99–11.73%	[111]
*Chlamydomonas reinhardtii*	Biofertilizer	250 mL Erlenmeyer flasks containing minimal media at 25 °C, 140 rpm, and 135 µmol photons m^−2^ s^−1^ continuous white light	Maximum uptake of nitrogen, phosphorus, and potassium increased by 185.17%, 119.36% and 78.04%, respectively	[112]
*Chlamydomonas reinhardtii* cc124	Biofertilizer	Bioreactor, Tris-acetate-phosphate, 25 °C, 16/8 h light/dark regime, white light, and shaker set at 180 rpm	Increased the plants’ shoot length, leaf size, fresh weight, number of flowers, and pigment content	[113]
*Chlamydomonas reinhardtii*	Biofertilizer	1 L flasks in a climatic chamber at a 16 h light/8 h dark regime at 22 °C/18 °C and light intensity µmol photons m^−2^ s^−1^ using Tris-acetate-phosphate	Increased the number of secondary roots, improved micro-nutrient accumulation in roots and shoots	[114]
*Chlamydomonas* sp.	Biofertilizer	Batch cultures incubated at 25 °C, in a 12:12 h light-and-dark cycle, and 130 µmol photons m^−2^ s^−1^	Increased growth, cell division, elongation, reproduction and respiration	[115]
*Chlamydomonas sajao*	Biofertilizer	Minimal medium, tubes incubated for 1 week at 25 °C at 5000-lx cool white light on a 16/8 h (light/dark) photo regime	Increased soil wet aggregate stability (33–77%)	[116]
*Chlamydomonas reinhardtii* cc-849	Bioplastic (PHB)	Tris-acetate-phosphate medium, continuous light of 90 µmol photons m^−2^ s^−1^ at 22 °C	126 nmol^−1^·min^−1^·mg prot^−1^	[118]
*Chlamydomonas reinhardtii* UVM4	Bioplastic (PHB)	Tris-acetate-phosphate medium, continuous light of 80 µmol photons m^−2^ s^−1^ 25 °C, and 120 rpm shaking	21.6 mg/g	[119]
*Chlamydomonas reinhardtii* C-9	Bioplastic (Cell-plastic)	80 L Photobioreactor, 25 °C, 150 µmol photons m^−2^ s^−1^, and 15,000 ppm CO_2_ in BG-11 medium	60% wt protein 6.6% wt carbohydrates 5.0% wt lipids	[121]
*Chlamydomonas* sp. JSC4	Biodiesel	Bioreactor, Tris-acetate-phosphate at 25 °C, and light intensity of 70 µmol photons m^−2^ s^−1^	96.2% oil recovery	[125]
*Chlamydomonas reinhardtii* UTEX 90	Bioethanol	Photo-bioreactor, Tris-acetate-phosphate medium, 96 h at 23 °C, and 130 rpm in a 2.5 L	235 mg/g algal biomass	[149]
*Chlamydomonas reinhardtii* UTEX 90	Bioethanol	Photobioreactor, 23 °C, Tris-acetate-phosphate medium, andcontinuous illumination at 450 µmol photons m^−2^ s^−1^	29.2% from algal biomass	[150]
*Chlamydomonas reinhardtii* UTEX 90	Bioethanol	Tris-acetate-phosphate medium, 25 °C, 100 µmol photons m^−2^ s^−1^, and 100 rpm	90–94% from algal biomass	[151]
*Chlamydomonas* sp. QWY37	Bioethanol	BG-11 medium, 27–30 °C, continuous supply of 2.5% CO_2_, and continuous illumination of 250 µmol photons m^−2^ s^−1^	61 g/L	[153]
*Chlamydomonas reinhardtii* cc124	Biogas	Tris-acetate-phosphate medium, 25 °C, and white light at 400 µmol photons m^−2^ s^−1^	587 mL of biogas per gram	[156]
*Chlamydomonas reinhardtii* CC-1690	Biogas	Photoautotrophically, glass bottles (max. capacity 3.5 L), and continuous white light at 300 µmol photons m^−2^ s^−1^	750 mL of biogas per gram	[158]
*Chlamydomonas reinhardtii* 6145	Biogas	Tris-acetate-phosphate medium, 12:8 light–dark cycles, 25 °C, and illumination of 36 µmol photons m^−2^ s^−1^	542 mL of biogas per gram	[160]
*Chlamydomonas reinhardtii* C137	Hydrogen	Anaerobic conditions involved using sulfur-starved culture under continuous illumination for up to 150 h	140 mL/L	[167]
*Chlamydomonas reinhardtii* 704	Hydrogen	Tris-acetate-phosphate medium, 25 °C, and white light at 12 µmol photons m^−2^ s^−1^ with acetic acid	65 mL/L	[168]
*Chlamydomonas* *reinhardtii* pgr5	Hydrogen	Tris-acetate-phosphate medium, 25 °C, white light at 90 µmol photons m^−2^ s^−1^, and constant agitation	65 mL/L	[172]
*Chlamydomonas reinhardtii* cc124	Hydrogen	Tris-acetate-phosphate medium, 25 °C, white light at 180 µmol photons m^−2^ s^−1^, and Argon atmosphere	3.26 mmol/L	[174]
*Chlamydomonas reinhardtii* HCR 89	Glycolate	Minimal-salts medium, 25 °C, 100 µmol photons m^−2^ s^−1^, 125 rpm, and 0.035% CO_2_	130 µmol/mg	[178]
*Chlamydomonas reinhardtii* Cia5	Glycolate	125 mL flasks of liquid Tris-acetate-phosphate medium on a shaker platform set at 100 rpm. Continuously illuminated at 65 µmol photons m^−2^ s^−1^, 25 °C, and no additional CO_2_ provided	0.3 g/L	[180]
*Chlamydomonas reinhardtii* AG 11–32b	Glycolate	Batch preculture at 20 °C, at a light intensity of 100 µmol photons m^−2^ s^−1^, Tris-phosphate minimal medium with Tris buffer (39.95 mM), and the addition of 3.08 µM FeSO_4_·7H_2_O plus 2.3 µM Na2-EDTA	41 mM	[181]
*Chlamydomonas reinhardtii* hpr1	Glycolate	Tris-acetate-phosphate at 25 °C under 80 µmol photons m^−2^ s^−1^ continuous light. Tris-minimal medium with aeration of 3% CO_2_	350 × 10^−6^ nmol/cell	[182]
*Chlamydomonas reinhardtii* UPN22	Bioisoprenoid	Tris-acetate-phosphate plus nitrate at 22 °C under 150 µmol photons m^−2^ s^−1^ continuous light and 120 rpm	152 mg/L	[184]
*Chlamydomonas reinhardtii* 137c	Hydroxyalkanoy- loxyalkanoate	Minimal high-salt medium with Spectinomycin at 25 °C under 50 µmol photons m^−2^ s^−1^ continuous light and 125 rpm	0.20 mg/L intracellular 0.16 mg/L extracellular	[185]
*Chlamydomonas reinhardtii* fap	7-heptadecene	Minimal high salt and Tris-acetate-phosphate in 24 deep well plates of 25 mL culture under 100 µmol photons m^−2^ s^−1^ at 25 °C. For day–night cycle experiment, autotrophically in 1L-photobiorectors in turbidostat mode	1.5% of total fatty acid methyl esters	[186]
*Chlamydomonas* sp. KR025878	ε-Polylysine	BG11 medium, under continuous illumination at 50 µmol photons m^−2^ s^−1^ at 27 °C with 100 rpm shaking. FeCl_3_ at 100 mg/L as flocculant and supplementation with lysine, aspartate, and 4 mM citric acid	2.24 g/L	[188]
*Chlamydomonas reinhardtii* UVM4	Polyamine (Cadaverine)	Mixotrophically in liquid or in solid Tris-acetate-phosphate medium and 250 µmol photons m^−2^ s^−1^ at 22 °C. Phototrophic in minimal medium supplied with 3–5% (*v*/*v*) CO_2_ enriched air	0.24 g/L after 9 days and maximal productivity of 0.1 g/L/d	[190]
*Chlamydomonas reinhardtii* ODC1	Polyamine (Putrescine)	Mixotrophic growth conditions on solid Tris-acetate phosphate, 350 µmol photons m^−2^ s^−1^ at 22 °C. For high-cell-density cultivations, 6x medium supplied with up to 10% (*v*/*v*) CO_2_-enriched air in 6-well plates	Maximum yield of 200 mg/L	[191]
*Chlamydomonas reinhardtii* TAI114	Protoporphyrin IX	Minimal-salts medium, 25 °C, 150 µmol photons m^−2^ s^−1^, 100 rpm, and 3–5% CO_2_	3–8% *w*/*w* of the dried biomass	[193]
*Chlamydomonas agloeformis* ChA	Antioxidants (flavonol)	Minimal-salts medium nitrate, 26 °C with 24:0 light–dark photoperiod, and a light intensity of 100 µmol photons m^−2^ s^−1^	203.80 ± 97.02 mg/100 g dried weight	
*Chlamydomonas reinhardtii* BKT	Antioxidants (Astaxanthin)	Tris-acetate-phosphate and 100–150 µmol photons m^−2^ s^−1^ at 25 °C. High-salt minimal media were used for photoautotrophic conditions. Growth was conducted using shaking flasks or stirring flasks	4.3 mg/L/day	[197]
*Chlamydomonas reinhardtii bkt5*	Antioxidants (Astaxanthin)	Tris-acetate-phosphate, 100 µmol photons m^−2^ s^−1^ at 25 °C. Growth in Multi-Cultivator MC-1000 (Photon Systems Instruments, Drásov, Czech Republic)	Up to 2.5 mg/g dry weight	[198]
*Chlamydomonas reinhardtii* ATG1-ATG8	Antioxidants (β-Carotene)	Tris-acetate-phosphate with Paromomycin 25 µg/m under continuous illumination of 100 µmol photons m^−2^ s^−1^ at 25 °C and shaken at 90 rpm	23.75 mg/g dry cell weight	[199]
*Chlamydomonas reinhardtii* VTC2	Antioxidants (vitamin C)	Mixotrophically in Tris-acetate-phosphate medium with arginine in 25–250 mL Erlenmeyer flasks on a rotatory shaker at 22 °C and 80 µmol photons m^−2^ s^−1^	Up to 1.3 mM	[201]
*Chlamydomonas reinhardtii*	Omega-3 fatty acids	Tris-acetate-phosphate medium, 100 rpm with ambient CO_2_ level, 23 °C, and 16:8 h alternating light–dark cycle with a photon irradiance of 100 µmol photons m^−2^ s^−1^	0.2–1.6 mg/g	[202]
*Chlamydomonas reinhardtii* CC-124	Sulphated polysaccharide	Tris-acetate-phosphate medium pH 7 and continuous illumination at 300 µmol photons m^−2^ s^−1^	130 mg/g	[203]
*Chlamydomonas reinhardtii* CR25	Therapeutic protein (ICAM)	Bioreactor, Tris-acetate-phosphate medium pH 7 with 15 μg/mL of Zeocin, and continuous illumination at 125 µmol photons m^−2^ s^−1^	46.6 mg/L	[207]
*Chlamydomonas reinhardtii* SRTA	Therapeutic protein (SARS-CoV-2)	Tris-acetate-phosphate medium pH 7 with 100 µg/mL spectinomycin and continuous illumination at 125 µmol photons m^−2^ s^−1^	11.2 ± 1.8 µg/L	[208]

## 6. Conclusions and Future Perspective

Throughout this review, various studies conducted with *Chlamydomonas* on bioremediation and bioproduct production have been presented. These studies demonstrate diverse applications across different fields. Traditional *Chlamydomonas* biotechnology has focused on identifying productive strains through bioprospecting and enhancing productivity using forward genetics. However, significant advancements in *Chlamydomonas* bioproductivity require integrating these methods with emerging molecular genetics tools. The production of bioproducts from *Chlamydomonas* faces numerous challenges, even at the laboratory level, which become more pronounced on an industrial scale. The high production costs of *Chlamydomonas*, which surpass those of raw materials, render the process economically unviable at present. Addressing these challenges is essential for advancing these processes and fully realizing their industrial potential.

We believe that a critical area for future development, due to its significant industrial and environmental impact, would be the simultaneous integration of these two aspects. Biomass obtained from bioremediation should be utilized for producing specific bioproducts of interest. As highlighted in this review, there have been some initial attempts in this direction, although development is hindered by substantial challenges. Overcoming these obstacles, such as the presence of harmful residues like xenobiotics and heavy metals in the biomass, difficulties in scaling up biomass production, high energy demands, and concerns about contamination by bacteria, fungi, and viruses, represents the primary limitation to the industrial utilization of *Chlamydomonas* for bioremediation and subsequent biomass reuse.

Additionally, to achieve industrial application of the *Chlamydomonas* laboratory-level studies presented in this review, it is crucial to conduct an economic analysis of their feasibility, which has not yet been undertaken. The studies discussed here reflect significant efforts towards future improvements and optimizations aimed at mitigating these issues and promoting a circular economy approach. Such advancements would not only minimize waste and encourage material reuse but also generate substantial environmental, economic, and industrial benefits.

## Figures and Tables

**Table 1:** Bioremediation characteristics and biomass production of different types of wastewaters using different strains of *Chlamydomonas*. Chemical oxygen demand (COD).

Microalgae	Wastewater Type	Cultivation/Growth Conditions	Bioremediation/Biomass Productivity	References
*Chlamydomonas reinhardtii (NIES-2235)*	Municipal Swine	Photobioreactor/28 ± 1 °C. Fluorescent lamps 80 μmol photons m^−2^s^−1^ and 16 h light/8 h dark for 1 week	Biomass: 187 mg dry weight/L	[39]
*Chlamydomonas debaryana IITRIND3*	Domestic Sewage Dairy	Photobioreactor/pH 7.4 at 27 °C and 140 rpm with white light illumination (200 mmol m^−1^s^−1^)	COD (105 mg L^−1^)/Biomass: 193 mg L^−1^/day	[40]
*Chlamydomonas debaryana AT24*	Swine wastewater	Photobioreactor/20–30 °C illuminated with white light (300–900 μmol photons m^−2^s^−1^). Air bubble (100 mL/min). 15 days cultivation	COD (29.8–46.0 mg L^−1^)	[41]
*Chlamydomonas reinhardtii*	Industrial	Photobioreactor/25 ± 1 °C. 120 μmol photons m^−2^s^−1^	N removal (55.8 mg L^−1^); P removal (17.4 mg L^−1^)/Biomass: 820 mg L^−1^/day	[42]
*Chlamydomonas mexicana*	Piggery wastewater	Batch/27 ± 1 °C and 150 rpm under continuous illumination for 20 days	N removal (23 mg L^−1^); P removal (5.1 mg L^−1^); Inorganic carbon (189 mg L^−1^); Calcium removal (17 mg L^−1^)/Biomass: 1.3 g L^−1^	[43]
*Chlamydomonas reinhardtii* sp.*ck*	Municipal	Photobioreactor/400 mL algae culture + Modified Provasoli-based minimal medium/100%–10% wastewater	Volatile solids (3.2–1.2 g L^−1^)/Biomass: 277 mg dry wight/L	[44]

## Data Availability

All data required to evaluate the conclusions of this paper are included in the main text.

## References

[1] Rani S., Gunjyal N., Ojha C.S.P., Singh R.P. (2021). Review of Challenges for Algae-Based Wastewater Treatment: Strain Selection, Wastewater Characteristics, Abiotic, and Biotic Factors. J. Hazard. Toxic Radioact. Waste.

[2] Falkowski P.G. (1994). The Role of Phytoplankton Photosynthesis in Global Biogeochemical Cycles. Photosynth. Res..

[3] Ochoa de Alda J.A.G., Esteban R., Diago M.L., Houmard J. (2014). The Plastid Ancestor Originated among One of the Major Cyanobacterial Lineages. Nat. Commun..

[4] Sibbald S.J., Archibald J.M. (2020). Genomic Insights into Plastid Evolution. Genome Biol. Evol..

[5] de Cassia Soares Brandão B., Oliveira C.Y.B., dos Santos E.P., de Abreu J.L., Oliveira D.W.S., da Silva S.M.B.C., Gálvez A.O. (2023). Microalgae-Based Domestic Wastewater Treatment: A Review of Biological Aspects, Bioremediation Potential, and Biomass Production with Biotechnological High-Value. Environ. Monit. Assess..

[6] Wang M., Ye X., Bi H., Shen Z. (2024). Microalgae Biofuels: Illuminating the Path to a Sustainable Future amidst Challenges and Opportunities. Biotechnol. Biofuels Bioprod..

[7] Gao S., Chen W., Cao S., Sun P., Gao X. (2024). Microalgae as Fishmeal Alternatives in Aquaculture: Current Status, Existing Problems, and Possible Solutions. Environ. Sci. Pollut. Res..

[8] Gupta A., Kang K., Pathania R., Saxton L., Saucedo B., Malik A., Torres-Tiji Y., Diaz C.J., Dutra Molino J.V., Mayfield S.P. (2024). Harnessing Genetic Engineering to Drive Economic Bioproduct Production in Algae. Front. Bioeng. Biotechnol..

[9] Udaypal, Goswami R.K., Mehariya S., Verma P. (2024). Advances in Microalgae-Based Carbon Sequestration: Current Status and Future Perspectives. Environ. Res..

[10] Fabris M., Abbriano R.M., Pernice M., Sutherland D.L., Commault A.S., Hall C.C., Labeeuw L., McCauley J.I., Kuzhiuparambil U., Ray P. (2020). Emerging Technologies in Algal Biotechnology: Toward the Establishment of a Sustainable, Algae-Based Bioeconomy. Front. Plant Sci..

[11] Sasso S., Stibor H., Mittag M., Grossman A.R. (2018). From Molecular Manipulation of Domesticated *Chlamydomonas reinhardtii* to Survival in Nature. Elife.

[12] Salomé P.A., Merchant S.S. (2019). A Series of Fortunate Events: Introducing *Chlamydomonas* as a Reference Organism. Plant Cell.

[13] Oz Yasar C., Fletcher L., Camargo-Valero M.A. (2023). Effect of Macronutrients (Carbon, Nitrogen, and Phosphorus) on the Growth of *Chlamydomonas reinhardtii* and Nutrient Recovery under Different Trophic Conditions. Environ. Sci. Pollut. Res..

[14] Goodenough U. (2023). The Chlamydomonas Sourcebook. Volume 1: Introduction to Chlamydomonas and Its Laboratory Use.

[15] Kselíková V., Singh A., Bialevich V., Čížková M., Bišová K. (2022). Improving Microalgae for Biotechnology—From Genetics to Synthetic Biology—Moving Forward but Not There Yet. Biotechnol. Adv..

[16] Calatrava V., Tejada-Jimenez M., Sanz-Luque E., Fernandez E., Galvan A., Llamas A. (2023). *Chlamydomonas reinhardtii*, a Reference Organism to Study Algal–Microbial Interactions: Why Can’t They Be Friends?. Plants.

[17] Llamas A., Leon-Miranda E., Tejada-Jimenez M. (2023). Microalgal and Nitrogen-Fixing Bacterial Consortia: From Interaction to Biotechnological Potential. Plants.

[18] Yang Y., Yu Q., Zhou R., Feng J., Zhang K., Li X., Ma X., Dietrich A.M. (2020). Occurrence of Free Amino Acids in the Source Waters of Zhejiang Province, China, and Their Removal and Transformation in Drinking Water Systems. Water.

[19] Ahamed A., Ge L., Zhao K., Veksha A., Bobacka J., Lisak G. (2021). Environmental Footprint of Voltammetric Sensors Based on Screen-Printed Electrodes: An Assessment towards “Green” Sensor Manufacturing. Chemosphere.

[20] Gaur N., Dutta D., Singh A., Dubey R., Kamboj D.V. (2022). Recent Advances in the Elimination of Persistent Organic Pollutants by Photocatalysis. Front. Environ. Sci..

[21] Wagner T.V., Rempe F., Hoek M., Schuman E., Langenhoff A. (2023). Key Constructed Wetland Design Features for Maximized Micropollutant Removal from Treated Municipal Wastewater: A Literature Study Based on 16 Indicator Micropollutants. Water Res..

[22] Morillas-España A., Lafarga T., Sánchez-Zurano A., Acién-Fernández F.G., González-López C. (2022). Microalgae Based Wastewater Treatment Coupled to the Production of High Value Agricultural Products: Current Needs and Challenges. Chemosphere.

[23] Elangovan B., Detchanamurthy S., Senthil Kumar P., Rajarathinam R., Deepa V.S. (2023). Biotreatment of Industrial Wastewater Using Microalgae: A Tool for a Sustainable Bioeconomy. Mol. Biotechnol..

[24] Talukdar A., Kundu P., Bhattacharya S., Dutta N. (2024). Microplastic Contamination in Wastewater: Sources, Distribution, Detection and Remediation through Physical and Chemical-Biological Methods. Sci. Total Environ..

[25] Krishnan R.Y., Manikandan S., Subbaiya R., Biruntha M., Govarthanan M., Karmegam N. (2021). Removal of Emerging Micropollutants Originating from Pharmaceuticals and Personal Care Products (PPCPs) in Water and Wastewater by Advanced Oxidation Processes: A Review. Environ. Technol. Innov..

[26] Singh A., Pal D.B., Mohammad A., Alhazmi A., Haque S., Yoon T., Srivastava N., Gupta V.K. (2022). Biological Remediation Technologies for Dyes and Heavy Metals in Wastewater Treatment: New Insight. Bioresour. Technol..

[27] Silva J.A. (2023). Wastewater Treatment and Reuse for Sustainable Water Resources Management: A Systematic Literature Review. Sustainability.

[28] Yadav G., Shanmugam S., Sivaramakrishnan R., Kumar D., Mathimani T., Brindhadevi K., Pugazhendhi A., Rajendran K. (2021). Mechanism and Challenges behind Algae as a Wastewater Treatment Choice for Bioenergy Production and Beyond. Fuel.

[29] Bhatia S.K., Mehariya S., Bhatia R.K., Kumar M., Pugazhendhi A., Awasthi M.K., Atabani A.E., Kumar G., Kim W., Seo S.O. (2021). Wastewater Based Microalgal Biorefinery for Bioenergy Production: Progress and Challenges. Sci. Total Environ..

[30] Razaviarani V., Arab G., Lerdwanawattana N., Gadia Y. (2023). Algal Biomass Dual Roles in Phycoremediation of Wastewater and Production of Bioenergy and Value-Added Products. Int. J. Environ. Sci. Technol..

[31] Dayana Priyadharshini S., Suresh Babu P., Manikandan S., Subbaiya R., Govarthanan M., Karmegam N. (2021). Phycoremediation of Wastewater for Pollutant Removal: A Green Approach to Environmental Protection and Long-Term Remediation. Environ. Pollut..

[32] Ibrahim F.G.G., Alonso Gómez V., Muñoz Torre R., de Godos Crespo I. (2023). Scale-down of High-Rate Algae Ponds Systems for Urban Wastewater Reuse. J. Water Process Eng..

[33] Villalba M.R., Cervera R., Sánchez J. (2023). Green Solutions for Urban Sustainability: Photobioreactors for Algae Cultivation on Façades and Artificial Trees. Buildings.

[34] Moreno Osorio J.H., Pollio A., Frunzo L., Lens P.N.L., Esposito G. (2021). A Review of Microalgal Biofilm Technologies: Definition, Applications, Settings and Analysis. Front. Chem. Eng..

[35] Vishwakarma J., Waghela B., Falcao B., Vavilala S.L. (2022). Algal Polysaccharide’s Potential to Combat Respiratory Infections Caused by *Klebsiella pneumoniae* and *Serratia marcescens* Biofilms. Appl. Biochem. Biotechnol..

[36] Leong Y.K., Chang J.S. (2020). Bioremediation of Heavy Metals Using Microalgae: Recent Advances and Mechanisms. Bioresour. Technol..

[37] Hoyos B.S., Hernandez-Tenorio F., Miranda A.M., Villanueva-Mejía D.F., Sáez A.A. (2023). Systematic Analysis of Genes Related to Selenium Bioaccumulation in Microalgae: A Review. Biology.

[38] Touliabah H.E.S., El-Sheekh M.M., Ismail M.M., El-Kassas H. (2022). A Review of Microalgae-and Cyanobacteria-Based Biodegradation of Organic Pollutants. Molecules.

[39] Toyama T., Kasuya M., Hanaoka T., Kobayashi N., Tanaka Y., Inoue D., Sei K., Morikawa M., Mori K. (2018). Growth Promotion of Three Microalgae, *Chlamydomonas reinhardtii*, *Chlorella vulgaris* and *Euglena gracilis*, by in Situ Indigenous Bacteria in Wastewater Effluent. Biotechnol. biofuels.

[40] Arora N., Patel A., Sartaj K., Pruthi P.A., Pruthi V. (2016). Bioremediation of Domestic and Industrial Wastewaters Integrated with Enhanced Biodiesel Production Using Novel Oleaginous Microalgae. Env. Sci. Pollut. Res..

[41] Hasan R. (2014). Bioremediation of Swine Wastewater and Biofuel Potential by Using *Chlorella vulgaris*, *Chlamydomonas reinhardtii*, and *Chlamydomonas debaryana*. J. Pet. Environ. Biotechnol..

[42] Kong Q.X., Li L., Martinez B., Chen P., Ruan R. (2010). Culture of Microalgae *Chlamydomonas reinhardtii* in Wastewater for Biomass Feedstock Production. Appl. Biochem. Biotechnol..

[43] Abou-Shanab R.A.I., Ji M.-K., Kim H.-C., Paeng K.-J., Jeon B.-H. (2013). Microalgal Species Growing on Piggery Wastewater as a Valuable Candidate for Nutrient Removal and Biodiesel Production. J. Environ. Manag..

[44] Klassen V., Blifernez-Klassen O., Bax J., Kruse O. (2020). Wastewater-Borne Microalga *Chlamydomonas* Sp.: A Robust Chassis for Efficient Biomass and Biomethane Production Applying Low-N Cultivation Strategy. Bioresour. Technol..

[45] Sasi P., Viswanathan A., Mechery J., Thomas D., Jacob J., Paulose S. (2020). Phycoremediation of Paper and Pulp Mill Effluent Using *Planktochlorella nurekis* and *Chlamydomonas reinhardtii*-A Comparative Study. J. Environ. Treat. Technol..

[46] Leong Y.K., Huang C.Y., Chang J.S. (2021). Pollution Prevention and Waste Phycoremediation by Algal-Based Wastewater Treatment Technologies: The Applications of High-Rate Algal Ponds (HRAPs) and Algal Turf Scrubber (ATS). J. Environ. Manag..

[47] Grönlund E., Hanaeus J., Johansson E., Falk S. (2010). Performance of an Experimental Wastewater Treatment High-Rate Algal Pond in Subarctic Climate. Water Environ. Res..

[48] de Godos I., Blanco S., García-Encina P.A., Becares E., Muñoz R. (2009). Long-Term Operation of High Rate Algal Ponds for the Bioremediation of Piggery Wastewaters at High Loading Rates. Bioresour. Technol..

[49] Bohutskyi P., Phan D., Spierling R.E., Kopachevsky A.M., Bouwer E.J., Lundquist T.J., Betenbaugh M.J. (2019). Production of Lipid-Containing Algal-Bacterial Polyculture in Wastewater and Biomethanation of Lipid Extracted Residues: Enhancing Methane Yield through Hydrothermal Pretreatment and Relieving Solvent Toxicity through Co-Digestion. Sci. Total Environ..

[50] Shen Y., Yu T., Xie Y., Chen J., Ho S.H., Wang Y., Huang F. (2019). Attached Culture of *Chlamydomonas* Sp. JSC4 for Biofilm Production and TN/TP/Cu(II) Removal. Biochem. Eng. J..

[51] Schaedig E., Cantrell M., Urban C., Zhao X., Greene D., Dancer J., Gross M., Sebesta J., Chou K.J., Grabowy J. (2023). Isolation of Phosphorus-Hyperaccumulating Microalgae from Revolving Algal Biofilm (RAB) Wastewater Treatment Systems. Front. Microbiol..

[52] de-Bashan L.E., Bashan Y. (2010). Immobilized Microalgae for Removing Pollutants: Review of Practical Aspects. Bioresour. Technol..

[53] Nazos T.T., Ghanotakis D.F. (2021). Biodegradation of Phenol by Alginate Immobilized *Chlamydomonas reinhardtii* Cells. Arch. Microbiol..

[54] Saavedra R., Muñoz R., Taboada M.E., Vega M., Bolado S. (2018). Comparative Uptake Study of Arsenic, Boron, Copper, Manganese and Zinc from Water by Different Green Microalgae. Bioresour. Technol..

[55] Xi Y., Han B., Kong F., You T., Bi R., Zeng X., Wang S., Jia Y. (2023). Enhancement of Arsenic Uptake and Accumulation in Green Microalga *Chlamydomonas reinhardtii* through Heterologous Expression of the Phosphate Transporter DsPht1. J. Hazard. Mater..

[56] Nam S.H., Kwak J.I., An Y.J. (2018). Assessing Applicability of the Paper-Disc Method Used in Combination with Flow Cytometry to Evaluate Algal Toxicity. Environ. Pollut..

[57] Ibuot A., Webster R.E., Williams L.E., Pittman J.K. (2020). Increased Metal Tolerance and Bioaccumulation of Zinc and Cadmium in *Chlamydomonas reinhardtii* Expressing a AtHMA4 C-Terminal Domain Protein. Biotechnol. Bioeng..

[58] Baselga-Cervera B., García-Balboa C., Díaz-Alejo H.M., Costas E., López-Rodas V. (2020). Rapid Colonization of Uranium Mining-Impacted Waters, the Biodiversity of Successful Lineages of Phytoplankton Extremophiles. Microb. Ecol..

[59] Balzano S., Sardo A., Blasio M., Chahine T.B., Dell’Anno F., Sansone C., Brunet C. (2020). Microalgal Metallothioneins and Phytochelatins and Their Potential Use in Bioremediation. Front. Microbiol..

[60] Zhang B., Tang Y., Yu F., Peng Z., Yao S., Deng X., Long H., Wang X., Huang K. (2023). Translatomics and Physiological Analyses of the Detoxification Mechanism of Green Alga *Chlamydomonas Reinhardtii* to Cadmium Toxicity. J. Hazard. Mater..

[61] Tang Y., Zhang B., Li Z., Deng P., Deng X., Long H., Wang X., Huang K. (2023). Overexpression of the Sulfate Transporter-Encoding *SULTR2* Increases Chromium Accumulation in *Chlamydomonas Reinhardtii*. Biotechnol. Bioeng..

[62] Li C., Li P., Fu H., Chen J., Ye M., Zhai S., Hu F., Zhang C., Ge Y., Fortin C. (2023). A Comparative Study of the Accumulation and Detoxification of Copper and Zinc in *Chlamydomonas Reinhardtii*: The Role of Extracellular Polymeric Substances. Sci. Total Environ..

[63] Millet R.T., Santos J.P., Slaveykova V.I. (2024). Exploring the Subcellular Distribution of Mercury in Green Alga *Chlamydomonas Reinhardtii* and Diatom *Cyclotella Meneghiniana*: A Comparative Study. Aquat. Toxicol..

[64] Sun D., Jiang Z., Yu H., Li Z., Zhang C., Ge Y. (2023). Assessment of Joint Toxicity of Arsenate and Lead by Multiple Endpoints in *Chlamydomonas reinhardtii*. Bull. Environ. Contam. Toxicol..

[65] Xu L., Zhao Z., Yan Z., Zhou G., Zhang W., Wang Y., Li X. (2022). Defense Pathways of *Chlamydomonas reinhardtii* under Silver Nanoparticle Stress: Extracellular Biosorption, Internalization and Antioxidant Genes. Chemosphere.

[66] Jin Z.P., Luo K., Zhang S., Zheng Q., Yang H. (2012). Bioaccumulation and Catabolism of Prometryne in Green Algae. Chemosphere.

[67] Wei S., Cao J., Ma X., Ping J., Zhang C., Ke T., Zhang Y., Tao Y., Chen L. (2020). The Simultaneous Removal of the Combined Pollutants of Hexavalent Chromium and O-Nitrophenol by *Chlamydomonas reinhardtii*. Ecotoxicol. Environ. Saf..

[68] Xiong J.Q., Kurade M.B., Abou-Shanab R.A.I., Ji M.K., Choi J., Kim J.O., Jeon B.H. (2016). Biodegradation of Carbamazepine Using Freshwater Microalgae *Chlamydomonas mexicana* and *Scenedesmus obliquus* and the Determination of Its Metabolic Fate. Bioresour. Technol..

[69] Wan L., Wu Y., Ding H., Zhang W. (2020). Toxicity, Biodegradation, and Metabolic Fate of Organophosphorus Pesticide Trichlorfon on the Freshwater Algae *Chlamydomonas reinhardtii*. J. Agric. Food Chem..

[70] Luo J., Deng J., Cui L., Chang P., Dai X., Yang C., Li N., Ren Z., Zhang X. (2020). The Potential Assessment of Green Alga *Chlamydomonas reinhardtii* CC-503 in the Biodegradation of Benz(a)Anthracene and the Related Mechanism Analysis. Chemosphere.

[71] Li S., Wang P., Zhang C., Zhou X., Yin Z., Hu T., Hu D., Liu C., Zhu L. (2020). Influence of Polystyrene Microplastics on the Growth, Photosynthetic Efficiency and Aggregation of Freshwater Microalgae *Chlamydomonas reinhardtii*. Sci. Total Environ..

[72] Carbó M., Chaturvedi P., Álvarez A., Pineda-Cevallos D., Ghatak A., González P.R., Cañal M.J., Weckwerth W., Valledor L. (2023). Ferroptosis Is the Key Cellular Process Mediating Bisphenol A Responses in *Chlamydomonas* and a Promising Target for Enhancing Microalgae-Based Bioremediation. J. Hazard. Mater..

[73] Hena S., Gutierrez L., Croué J.P. (2021). Removal of Pharmaceutical and Personal Care Products (PPCPs) from Wastewater Using Microalgae: A Review. J. Hazard. Mater..

[74] Guo W.Q., Zheng H.S., Li S., Du J.S., Feng X.C., Yin R.L., Wu Q.L., Ren N.Q., Chang J.S. (2016). Removal of Cephalosporin Antibiotics 7-ACA from Wastewater during the Cultivation of Lipid-Accumulating Microalgae. Bioresour. Technol..

[75] Xiong J.Q., Kurade M.B., Jeon B.H. (2017). Ecotoxicological Effects of Enrofloxacin and Its Removal by Monoculture of Microalgal Species and Their Consortium. Environ. Pollut..

[76] Zhou G.-J., Ying G.-G., Liu S., Zhou L.-J., Chen Z.-F., Peng F.-Q. (2014). Simultaneous Removal of Inorganic and Organic Compounds in Wastewater by Freshwater Green Microalgae. Environ. Sci. Process Impacts.

[77] Li Z., Dong S., Huang F., Lin L., Hu Z., Zheng Y. (2022). Toxicological Effects of Microplastics and Sulfadiazine on the Microalgae *Chlamydomonas reinhardtii*. Front. Microbiol..

[78] Seoane M., Conde-Pérez K., Esperanza M., Cid Á., Rioboo C. (2023). Unravelling Joint Cytotoxicity of Ibuprofen and Oxytetracycline on *Chlamydomonas Reinhardtii* Using a Programmed Cell Death-Related Biomarkers Panel. Aquat. Toxicol..

[79] Hom-Diaz A., Jaén-Gil A., Rodríguez-Mozaz S., Barceló D., Vicent T., Blánquez P. (2022). Insights into Removal of Antibiotics by Selected Microalgae (*Chlamydomonas reinhardtii*, *Chlorella sorokiniana*, *Dunaliella tertiolecta* and *Pseudokirchneriella subcapitata*). Algal Res..

[80] Hom-Diaz A., Llorca M., Rodríguez-Mozaz S., Vicent T., Barceló D., Blánquez P. (2015). Microalgae Cultivation on Wastewater Digestate: β-Estradiol and 17α-Ethynylestradiol Degradation and Transformation Products Identification. J. Environ. Manag..

[81] Liakh I., Harshkova D., Hrouzek P., Bišová K., Aksmann A., Wielgomas B. (2023). Green Alga *Chlamydomonas Reinhardtii* Can Effectively Remove Diclofenac from the Water Environment—A New Perspective on Biotransformation. J. Hazard. Mater..

[82] Stravs M.A., Pomati F., Hollender J. (2017). Exploring Micropollutant Biotransformation in Three Freshwater Phytoplankton Species. Environ. Sci. Process Impacts.

[83] Otto B., Beuchel C., Liers C., Reisser W., Harms H., Schlosser D. (2015). Laccase-like Enzyme Activities from Chlorophycean Green Algae with Potential for Bioconversion of Phenolic Pollutants. FEMS Microbiol. Lett..

[84] Míguez L., Esperanza M., Seoane M., Cid Á. (2021). Assessment of Cytotoxicity Biomarkers on the Microalga *Chlamydomonas Reinhardtii* Exposed to Emerging and Priority Pollutants. Ecotoxicol. Environ. Saf..

[85] Yadav N., Ahn H.J., Kurade M.B., Ahn Y., Park Y.K., Khan M.A., Salama E.S., Li X., Jeon B.H. (2023). Fate of Five Bisphenol Derivatives in *Chlamydomonas Mexicana*: Toxicity, Removal, Biotransformation and Microalgal Metabolism. J. Hazard. Mater..

[86] Yoshida S., Hiraga K., Takehana T., Taniguchi I., Yamaji H., Maeda Y., Toyohara K., Miyamoto K., Kimura Y., Oda K. (2016). A Bacterium That Degrades and Assimilates Poly(Ethylene Terephthalate). Science.

[87] Di Rocco G., Taunt H.N., Berto M., Jackson H.O., Piccinini D., Carletti A., Scurani G., Braidi N., Purton S. (2023). A PETase Enzyme Synthesised in the Chloroplast of the Microalga *Chlamydomonas reinhardtii* Is Active against Post-Consumer Plastics. Sci. Rep..

[88] de Carpentier F., Maes A., Marchand C.H., Chung C., Durand C., Crozet P., Lemaire S.D., Danon A. (2022). How Abiotic Stress-Induced Socialization Leads to the Formation of Massive Aggregates in *Chlamydomonas*. Plant Physiol..

[89] Zhang X., Zhang Y., Chen Z., Gu P., Li X., Wang G. (2023). Exploring Cell Aggregation as a Defense Strategy against Perchlorate Stress in *Chlamydomonas reinhardtii* through Multi-Omics Analysis. Sci. Total Environ..

[90] Vieira M.V., Pastrana L.M., Fuciños P. (2020). Microalgae Encapsulation Systems for Food, Pharmaceutical and Cosmetics Applications. Mar. Drugs.

[91] Chen Z., Osman A.I., Rooney D.W., Oh W.D., Yap P.S. (2023). Remediation of Heavy Metals in Polluted Water by Immobilized Algae: Current Applications and Future Perspectives. Sustainability.

[92] Lee H., Jeong D., Im S.J., Jang A. (2020). Optimization of Alginate Bead Size Immobilized with Chlorella Vulgaris and *Chlamydomonas reinhardtii* for Nutrient Removal. Bioresour. Technol..

[93] Erkaya I.A., Arica M.Y., Akbulut A., Bayramoglu G. (2014). Biosorption of Uranium(VI) by Free and Entrapped *Chlamydomonas Reinhardtii*: Kinetic, Equilibrium and Thermodynamic Studies. J. Radioanal. Nucl. Chem..

[94] Nakanishi A., Sakihama Y., Ozawa N. (2021). Improvement of Growth of *Chlamydomonas reinhardtii* in CO_2_—Stepwisely Aerating Condition. J. Appl. Biotechnol. Rep..

[95] Hariz H.B., Takriff M.S., Mohd Yasin N.H., Ba-Abbad M.M., Mohd Hakimi N.I.N. (2019). Potential of the Microalgae-Based Integrated Wastewater Treatment and CO_2_ Fixation System to Treat Palm Oil Mill Effluent (POME) by Indigenous Microalgae; *Scenedesmus* sp. and *Chlorella* sp.. J. Water Process Eng..

[96] Choi H.I., Hwang S.W., Kim J., Park B., Jin E.S., Choi I.G., Sim S.J. (2021). Augmented CO_2_ Tolerance by Expressing a Single H+-Pump Enables Microalgal Valorization of Industrial Flue Gas. Nat. Commun..

[97] Amin M., Tahir F., Ashfaq H., Akbar I., Razzaque N., Haider M.N., Xu J., Zhu H., Wang N., Shahid A. (2022). Decontamination of Industrial Wastewater Using Microalgae Integrated with Biotransformation of the Biomass to Green Products. Energy Nexus.

[98] Ahmad A., Hassan S.W., Banat F. (2022). An Overview of Microalgae Biomass as a Sustainable Aquaculture Feed Ingredient: Food Security and Circular Economy. Bioengineered.

[99] Ma X., Mi Y., Zhao C., Wei Q. (2022). A Comprehensive Review on Carbon Source Effect of Microalgae Lipid Accumulation for Biofuel Production. Sci. Total Environ..

[100] Cheng C.L., Lo Y.C., Huang K.L., Nagarajan D., Chen C.Y., Lee D.J., Chang J.S. (2022). Effect of PH on Biomass Production and Carbohydrate Accumulation of *Chlorella vulgaris* JSC-6 under Autotrophic, Mixotrophic, and Photoheterotrophic Cultivation. Bioresour. Technol..

[101] Beigbeder J.B., Lavoie J.M. (2022). Effect of Photoperiods and CO_2_ Concentrations on the Cultivation of Carbohydrate-Rich *P. Kessleri* Microalgae for the Sustainable Production of Bioethanol. J. CO2 Util..

[102] Salman J.M., Grmasha R.A., Stenger-Kovács C., Lengyel E., Al-sareji O.J., AL-Cheban A.M.A.A., Meiczinger M. (2023). Influence of Magnesium Concentrations on the Biomass and Biochemical Variations in the Freshwater Algae, *Chlorella vulgaris*. Heliyon.

[103] Fields F.J., Ostrand J.T., Mayfield S.P. (2018). Fed-Batch Mixotrophic Cultivation of *Chlamydomonas reinhardtii* for High-Density Cultures. Algal Res..

[104] Jin H., Chuai W., Li K., Hou G., Wu M., Chen J., Wang H., Jia J., Han D., Hu Q. (2021). Ultrahigh-Cell-Density Heterotrophic Cultivation of the Unicellular Green Alga *Chlorella Sorokiniana* for Biomass Production. Biotechnol. Bioeng..

[105] Leong Y.K., Chang J.S. (2023). Microalgae-Based Biochar Production and Applications: A Comprehensive Review. Bioresour. Technol..

[106] Fan X., Du C., Zhou L., Fang Y., Zhang G., Zou H., Yu G., Wu H. (2024). Biochar from Phytoremediation Plant Residues: A Review of Its Characteristics and Potential Applications. Environ. Sci. Pollut. Res..

[107] Torri C., Samorì C., Adamiano A., Fabbri D., Faraloni C., Torzillo G. (2011). Preliminary Investigation on the Production of Fuels and Bio-Char from *Chlamydomonas reinhardtii* Biomass Residue after Bio-Hydrogen Production. Bioresour. Technol..

[108] Gan Y.Y., Ong H.C., Show P.L., Ling T.C., Chen W.H., Yu K.L., Abdullah R. (2018). Torrefaction of Microalgal Biochar as Potential Coal Fuel and Application as Bio-Adsorbent. Energy Convers. Manag..

[109] Zheng H., Guo W., Li S., Chen Y., Wu Q., Feng X., Yin R., Ho S.H., Ren N., Chang J.S. (2017). Adsorption of P-Nitrophenols (PNP) on Microalgal Biochar: Analysis of High Adsorption Capacity and Mechanism. Bioresour. Technol..

[110] Miranda A.M., Hernandez-Tenorio F., Villalta F., Vargas G.J., Sáez A.A. (2024). Advances in the Development of Biofertilizers and Biostimulants from Microalgae. Biology.

[111] Sido M.Y., Tian Y., Wang X., Wang X. (2022). Application of Microalgae *Chlamydomonas applanata* M9V and *Chlorella vulgaris* S3 for Wheat Growth Promotion and as Urea Alternatives. Front. Microbiol..

[112] Mutale-Joan C., Redouane B., Najib E., Yassine K., Lyamlouli K., Laila S., Zeroual Y., Hicham E.A. (2020). Screening of Microalgae Liquid Extracts for Their Bio Stimulant Properties on Plant Growth, Nutrient Uptake and Metabolite Profile of *Solanum lycopersicum* L.. Sci. Rep..

[113] Gitau M.M., Farkas A., Balla B., Ördög V., Futó Z., Maróti G. (2021). Strain-Specific Biostimulant Effects of *Chlorella* and *Chlamydomonas* Green Microalgae on *Medicago truncatula*. Plants.

[114] Martini F., Beghini G., Zanin L., Varanini Z., Zamboni A., Ballottari M. (2021). The Potential Use of *Chlamydomonas reinhardtii* and *Chlorella sorokiniana* as Biostimulants on Maize Plants. Algal Res..

[115] Stirk W.A., Ördög V., Staden J.V., Jäger K. (2002). Cytokinin-and Auxin-like Activity in Cyanophyta and Microalgae. J. Appl. Phycol..

[116] Metting B. (1986). Population Dynamics of *Chlamydomonas sajao* and Its Influence on Soil Aggregate Stabilization in the Field. Appl. Environ. Microbiol..

[117] Arora Y., Sharma S., Sharma V. (2023). Microalgae in Bioplastic Production: A Comprehensive Review. Arab. J. Sci. Eng..

[118] Chaogang W., Zhangli H., Anping L., Baohui J. (2010). Biosynthesis of Poly-3-Hydroxybutyrate (PHB) in the Transgenic Green Alga *Chlamydomonas reinhardtii*. J. Phycol..

[119] Hao Z., Songlin M., Xiaotan D., Ru C., Han L., Zhanyou C., Song X., Yonghua L.-B., Fantao K. (2024). Harnessing Algal Peroxisomes for Efficient Poly Hydroxybutyrate Production. ACS Sustain. Chem. Eng..

[120] Kato N. (2019). Production of Crude Bioplastic-Beads with Microalgae: Proof-of-Concept. Bioresour. Technol. Rep..

[121] Nakanishi A., Nemoto S., Yamamoto N., Iritani K., Watanabe M. (2023). Identification of Cell-Attachment Factors Derived from Green Algal Cells Disrupted by Sonication in Fabrication of Cell Plastics. Bioengineering.

[122] Chaos-Hernández D., Reynel-Ávila H.E., Bonilla-Petriciolet A., Villalobos-Delgado F.J. (2023). Extraction Methods of Algae Oils for the Production of Third Generation Biofuels—A Review. Chemosphere.

[123] Daneshvar E., Sik Ok Y., Tavakoli S., Sarkar B., Shaheen S.M., Hong H., Luo Y., Rinklebe J., Song H., Bhatnagar A. (2021). Insights into Upstream Processing of Microalgae: A Review. Bioresour. Technol..

[124] Sivaramakrishnan R., Suresh S., Kanwal S., Ramadoss G., Ramprakash B., Incharoensakdi A. (2022). Microalgal Biorefinery Concepts’ Developments for Biofuel and Bioproducts: Current Perspective and Bottlenecks. Int. J. Mol. Sci..

[125] Chen C.L., Huang C.C., Ho K.C., Hsiao P.X., Wu M.S., Chang J.S. (2015). Biodiesel Production from Wet Microalgae Feedstock Using Sequential Wet Extraction/Transesterification and Direct Transesterification Processes. Bioresour. Technol..

[126] Kao P.H., Ng I.S. (2017). CRISPRi Mediated Phosphoenolpyruvate Carboxylase Regulation to Enhance the Production of Lipid in *Chlamydomonas reinhardtii*. Bioresour. Technol..

[127] Rengel R., Smith R.T., Haslam R.P., Sayanova O., Vila M., León R. (2018). Overexpression of Acetyl-CoA Synthetase (ACS) Enhances the Biosynthesis of Neutral Lipids and Starch in the Green Microalga *Chlamydomonas reinhardtii*. Algal Res..

[128] Kong F., Liang Y., Légeret B., Beyly-Adriano A., Blangy S., Haslam R.P., Napier J.A., Beisson F., Peltier G., Li-Beisson Y. (2017). *Chlamydomonas* Carries out Fatty Acid β-Oxidation in Ancestral Peroxisomes Using a Bona Fide Acyl-CoA Oxidase. Plant J..

[129] Shin Y.S., Jeong J., Nguyen T.H.T., Kim J.Y.H., Jin E.S., Sim S.J. (2019). Targeted Knockout of Phospholipase A2 to Increase Lipid Productivity in *Chlamydomonas Reinhardtii* for Biodiesel Production. Bioresour. Technol..

[130] Huang L.-F., Lin J.-Y., Pan K.-Y., Huang C.-K., Chu Y.-K. (2015). Overexpressing Ferredoxins in *Chlamydomonas reinhardtii* Increase Starch and Oil Yields and Enhance Electric Power Production in a Photo Microbial Fuel Cell. Int. J. Mol. Sci..

[131] Pancha I., Chokshi K., Tanaka K., Imamura S. (2020). Microalgal Target of Rapamycin (TOR): A Central Regulatory Hub for Growth, Stress Response and Biomass Production. Plant Cell Physiol..

[132] Tan K.W.M., Lee Y.K. (2017). Expression of the Heterologous *Dunaliella tertiolecta* Fatty Acyl-ACP Thioesterase Leads to Increased Lipid Production in *Chlamydomonas reinhardtii*. J. Biotechnol..

[133] Zhu Z., Yuan G., Fan X., Fan Y., Yang M., Yin Y., Liu J., Liu Y., Cao X., Tian J. (2018). The Synchronous TAG Production with the Growth by the Expression of Chloroplast Transit Peptide-Fused ScPDAT in *Chlamydomonas reinhardtii*. Biotechnol. Biofuels.

[134] Iskandarov U., Sitnik S., Shtaida N., Didi-Cohen S., Leu S., Khozin-Goldberg I., Cohen Z., Boussiba S. (2016). Cloning and Characterization of a GPAT-like Gene from the Microalga *Lobosphaera incisa* (Trebouxiophyceae): Overexpression in *Chlamydomonas reinhardtii* Enhances TAG Production. J. Appl. Phycol..

[135] Li Y., Han D., Hu G., Sommerfeld M., Hu Q. (2010). Inhibition of Starch Synthesis Results in Overproduction of Lipids in *Chlamydomonas reinhardtii*. Biotechnol. Bioeng..

[136] Kato Y., Oyama T., Inokuma K., Vavricka C.J., Matsuda M., Hidese R., Satoh K., Oono Y., Chang J.S., Hasunuma T. (2021). Enhancing Carbohydrate Repartitioning into Lipid and Carotenoid by Disruption of Microalgae Starch Debranching Enzyme. Commun. Biol..

[137] Khoo K.S., Ahmad I., Chew K.W., Iwamoto K., Bhatnagar A., Show P.L. (2023). Enhanced Microalgal Lipid Production for Biofuel Using Different Strategies Including Genetic Modification of Microalgae: A Review. Prog. Energy Combust. Sci..

[138] Zheng S., Zou S., Wang H., Feng T., Sun S., Chen H., Wang Q. (2022). Reducing Culture Medium Nitrogen Supply Coupled with Replenishing Carbon Nutrient Simultaneously Enhances the Biomass and Lipid Production of *Chlamydomonas reinhardtii*. Front. Microbiol..

[139] Goodenough U., Blaby I., Casero D., Gallaher S.D., Goodson C., Johnson S., Lee J.H., Merchant S.S., Pellegrini M., Roth R. (2014). The Path to Triacylglyceride Obesity in the Sta6 Strain of *Chlamydomonas reinhardtii*. Eukaryot. Cell.

[140] Kim J.H., Ahn J.W., Park E.J., Choi J.L. (2023). Overexpression of S-Adenosylmethionine Synthetase in Recombinant *Chlamydomonas* for Enhanced Lipid Production. J. Microbiol. Biotechnol..

[141] Maltsev Y., Kulikovskiy M., Maltseva S. (2023). Nitrogen and Phosphorus Stress as a Tool to Induce Lipid Production in Microalgae. Microb. Cell Fact..

[142] Gonzalez D.I., Ynalvez R.A. (2023). Comparison of the Effects of Nitrogen-, Sulfur- and Combined Nitrogen- and Sulfur-Deprivations on Cell Growth, Lipid Bodies and Gene Expressions in *Chlamydomonas reinhardtii* Cc5373-Sta6. BMC Biotechnol..

[143] Salama E.S., Kim H.C., Abou-Shanab R.A.I., Ji M.K., Oh Y.K., Kim S.H., Jeon B.H. (2013). Biomass, Lipid Content, and Fatty Acid Composition of Freshwater *Chlamydomonas mexicana* and *Scenedesmus obliquus* Grown under Salt Stress. Bioprocess Biosyst. Eng..

[144] James G.O., Hocart C.H., Hillier W., Price G.D., Djordjevic M.A. (2013). Temperature Modulation of Fatty Acid Profiles for Biofuel Production in Nitrogen Deprived *Chlamydomonas reinhardtii*. Bioresour. Technol..

[145] Zhao J., Ge Y., Liu K., Yamaoka Y., Zhang D., Chi Z., Akkaya M., Kong F. (2023). Overexpression of a *MYB1* Transcription Factor Enhances Triacylglycerol and Starch Accumulation and Biomass Production in the Green Microalga *Chlamydomonas reinhardtii*. J. Agric. Food Chem..

[146] Singh P., Kumari S., Guldhe A., Misra R., Rawat I., Bux F. (2016). Trends and Novel Strategies for Enhancing Lipid Accumulation and Quality in Microalgae. Renew. Sustain. Energy Rev..

[147] Figueroa-Torres G.M., Pittman J.K., Theodoropoulos C. (2021). Optimisation of Microalgal Cultivation via Nutrient-Enhanced Strategies: The Biorefinery Paradigm. Biotechnol. Biofuels.

[148] Poulhazan A., Arnold A.A., Mentink-Vigier F., Muszyński A., Azadi P., Halim A., Vakhrushev S.Y., Joshi H.J., Wang T., Warschawski D.E. (2024). Molecular-Level Architecture of *Chlamydomonas reinhardtii’s* Glycoprotein-Rich Cell Wall. Nat. Commun..

[149] Choi S.P., Nguyen M.T., Sim S.J. (2010). Enzymatic Pretreatment of *Chlamydomonas Reinhardtii* Biomass for Ethanol Production. Bioresour. Technol..

[150] Nguyen M.T., Choi S.P., Lee J., Lee J.H., Sim S.J. (2009). Hydrothermal Acid Pretreatment of *Chlamydomonas reinhardtii* Biomass for Ethanol Production. J. Microbiol. Biotechnol..

[151] Ivanov I.N., Zachleder V., Vítová M., Barbosa M.J., Bišová K. (2021). Starch Production in *Chlamydomonas reinhardtii* through Supraoptimal Temperature in a Pilot-Scale Photobioreactor. Cells.

[152] De Marco M.A., Curatti L., Martínez-Noël G.M.A. (2024). High Auxin Disrupts Expression of Cell-Cycle Genes, Arrests Cell Division and Promotes Accumulation of Starch in *Chlamydomonas reinhardtii*. Algal Res..

[153] Qu W., Loke Show P., Hasunuma T., Ho S.H. (2020). Optimizing Real Swine Wastewater Treatment Efficiency and Carbohydrate Productivity of Newly Microalga Chlamydomonas Sp. QWY37 Used for Cell-Displayed Bioethanol Production. Bioresour. Technol..

[154] Kunatsa T., Xia X. (2022). A Review on Anaerobic Digestion with Focus on the Role of Biomass Co-Digestion, Modelling and Optimisation on Biogas Production and Enhancement. Bioresour. Technol..

[155] Jha P., Ghosh S., Panja A., Kumar V., Singh A.K., Prasad R. (2023). Microalgae and Biogas: A Boon to Energy Sector. Environ. Sci. Pollut. Res..

[156] Mussgnug J.H., Klassen V., Schlüter A., Kruse O. (2010). Microalgae as Substrates for Fermentative Biogas Production in a Combined Biorefinery Concept. J. Biotechnol..

[157] Veerabadhran M., Gnanasekaran D., Wei J., Yang F. (2021). Anaerobic Digestion of Microalgal Biomass for Bioenergy Production, Removal of Nutrients and Microcystin: Current Status. J. Appl. Microbiol..

[158] Klassen V., Blifernez-Klassen O., Wibberg D., Winkler A., Kalinowski J., Posten C., Kruse O. (2017). Highly Efficient Methane Generation from Untreated Microalgae Biomass. Biotechnol. Biofuels.

[159] Zabed H.M., Akter S., Yun J., Zhang G., Zhang Y., Qi X. (2020). Biogas from Microalgae: Technologies, Challenges and Opportunities. Renew. Sustain. Energy Rev..

[160] Fernández-Rodríguez M.J., de la Lama-Calvente D., Jiménez-Rodríguez A., Borja R., Rincón-Llorente B. (2019). Influence of the Cell Wall of *Chlamydomonas reinhardtii* on Anaerobic Digestion Yield and on Its Anaerobic Co-Digestion with a Carbon-Rich Substrate. Process Saf. Environ. Prot..

[161] Barros R., Raposo S., Morais E.G., Rodrigues B., Afonso V., Gonçalves P., Marques J., Cerqueira P.R., Varela J., Teixeira M.R. (2022). Biogas Production from Microalgal Biomass Produced in the Tertiary Treatment of Urban Wastewater: Assessment of Seasonal Variations. Energies.

[162] Nirmala N., Praveen G., AmitKumar S., SundarRajan P.S., Baskaran A., Priyadharsini P., SanjayKumar S.P., Dawn S.S., Pavithra K.G., Arun J. (2023). A Review on Biological Biohydrogen Production: Outlook on Genetic Strain Enhancements, Reactor Model and Techno-Economics Analysis. Sci. Total Environ..

[163] Xu X., Zhou Q., Yu D. (2022). The Future of Hydrogen Energy: Bio-Hydrogen Production Technology. Int. J. Hydrog. Energy.

[164] King S.J., Jerkovic A., Brown L.J., Petroll K., Willows R.D. (2022). Synthetic Biology for Improved Hydrogen Production in *Chlamydomonas reinhardtii*. Microb. Biotechnol..

[165] Frenkel A.W. (1951). Hydrogen Evolution by the Flagellate Green Alga, *Chlamydomonas moewusii*. Arch Biochem Biophys.

[166] Ben-Amotz A., Erbes D.L., Riederer-Henderson M.A., Peavey D.G., Gibbs M. (1975). H2 Metabolism in Photosynthetic Organisms: I. Dark H2 Evolution and Uptake by Algae and Mosses. Plant Physiol.

[167] Melis A., Zhang L., Forestier M., Ghirardi M.L., Seibert M. (2000). Sustained Photobiological Hydrogen Gas Production upon Reversible Inactivation of Oxygen Evolution in the Green Alga *Chlamydomonas reinhardtii*. Plant Physiol..

[168] Jurado-Oller J.L., Dubini A., Galván A., Fernández E., González-Ballester D. (2015). Low Oxygen Levels Contribute to Improve Photohydrogen Production in Mixotrophic Non-Stressed *Chlamydomonas* Cultures. Biotechnol. Biofuels.

[169] Elman T., Yacoby I. (2022). A Two-Phase Protocol for Ambient Hydrogen Production Using *Chlamydomonas reinhardtii*. STAR Protoc..

[170] Fakhimi N., Gonzalez-Ballester D., Fernández E., Galván A., Dubini A. (2020). Algae-Bacteria Consortia as a Strategy to Enhance H_2_ Production. Cells.

[171] Elman T., Schweitzer S., Shahar N., Swartz J., Yacoby I. (2020). Engineered Clostridial [FeFe]-Hydrogenase Shows Improved O2 Tolerance in *Chlamydomonas reinhardtii*. Int. J. Hydrog. Energy.

[172] Nagy V., Podmaniczki A., Vidal-Meireles A., Kuntam S., Herman É., Kovács L., Tóth D., Scoma A., Tóth S.Z. (2021). Thin Cell Layer Cultures of *Chlamydomonas reinhardtii* L159I-N230Y, *Pgrl1* and *Pgr5* Mutants Perform Enhanced Hydrogen Production at Sunlight Intensity. Bioresour. Technol..

[173] Milrad Y., Schweitzer S., Feldman Y., Yacoby I. (2018). Green Algal Hydrogenase Activity Is Outcompeted by Carbon Fixation before Inactivation by Oxygen Takes Place. Plant Physiol..

[174] Kosourov S., Nagy V., Shevela D., Jokel M., Messinger J., Allahverdiyeva Y. (2020). Water Oxidation by Photosystem II Is the Primary Source of Electrons for Sustained H2 Photoproduction in Nutrient-Replete Green Algae. PNAS.

[175] Kanygin A., Milrad Y., Thummala C., Reifschneider K., Baker P., Marco P., Yacoby I., Redding K.E. (2020). Rewiring Photosynthesis: A Photosystem I-Hydrogenase Chimera That Makes H_2_: In Vivo. Energy Environ. Sci..

[176] Lakatos G., Balogh D., Farkas A., Ördög V., Nagy P., Bíró T., Maróti G. (2017). Factors Influencing Algal Photobiohydrogen Production in Algal-Bacterial Co-Cultures. Algal Res.

[177] Masi A., Leonelli F., Scognamiglio V., Gasperuzzo G., Antonacci A., Terzidis M.A. (2023). *Chlamydomonas reinhardtii*: A Factory of Nutraceutical and Food Supplements for Human Health. Molecules.

[178] Nakamura Y. (2005). Disruption of the Glycolate Dehydrogenase Gene in the High-CO_2_-Requiring Mutant HCR89 of *Chlamydomonas reinhardtii*. Can. J. Bot..

[179] Wang Y., Stessman D.J., Spalding M.H. (2015). The CO2 Concentrating Mechanism and Photosynthetic Carbon Assimilation in Limiting CO_2_: How *Chlamydomonas* Works against the Gradient. Plant J..

[180] Yun E.J., Zhang G.-C., Atkinson C., Lane S., Liu J.-J., Ort D.R., Jin Y.-S. (2021). Glycolate Production by a *Chlamydomonas Reinhardtii* Mutant Lacking Carbon-Concentrating Mechanism. J. Biotechnol..

[181] Taubert A., Jakob T., Wilhelm C. (2019). Glycolate from Microalgae: An Efficient Carbon Source for Biotechnological Applications. Plant Biotechnol. J..

[182] Zabaleta E., Wang Y., Zhao L., Shi M. (2021). Identification and Characterization of Genes Encoding the Hydroxypyruvate Reductases in *Chlamydomonas* Reveal Their Distinct Roles in Photorespiration. Front. Plant Sci..

[183] Lauersen K.J. (2019). Eukaryotic Microalgae as Hosts for Light-Driven Heterologous Isoprenoid Production. Planta.

[184] Yahya R.Z., Wellman G.B., Overmans S., Lauersen K.J. (2023). Engineered Production of Isoprene from the Model Green Microalga *Chlamydomonas reinhardtii*. Metab. Eng. Commun..

[185] Miró-Vinyals B., Artigues M., Wostrikoff K., Monte E., Broto-Puig F., Leivar P., Planas A. (2023). Chloroplast Engineering of the Green Microalgae *Chlamydomonas reinhardtii* for the Production of HAA, the Lipid Moiety of Rhamnolipid Biosurfactants. N. Biotechnol..

[186] Moulin S.L.Y., Beyly-Adriano A., Cuiné S., Blangy S., Légeret B., Floriani M., Burlacot A., Sorigué D., Samire P.P., Li-Beisson Y. (2021). Fatty Acid Photodecarboxylase Is an Ancient Photoenzyme That Forms Hydrocarbons in the Thylakoids of Algae. Plant Physiol..

[187] Salma-Ancane K., Sceglovs A., Tracuma E., Wychowaniec J.K., Aunina K., Ramata-Stunda A., Nikolajeva V., Loca D. (2022). Effect of Crosslinking Strategy on the Biological, Antibacterial and Physicochemical Performance of Hyaluronic Acid and ε-Polylysine Based Hydrogels. Int. J. Biol. Macromol..

[188] Sivaramakrishnan R., Suresh S., Incharoensakdi A. (2019). *Chlamydomonas* Sp. as Dynamic Biorefinery Feedstock for the Production of Methyl Ester and ε-Polylysine. Bioresour. Technol..

[189] Lee J.A., Kim J.Y., Ahn J.H., Ahn Y.-J., Lee S.Y. (2023). Current Advancements in the Bio-Based Production of Polyamides. Trends Chem..

[190] Freudenberg R.A., Baier T., Einhaus A., Wobbe L., Kruse O. (2021). High Cell Density Cultivation Enables Efficient and Sustainable Recombinant Polyamine Production in the Microalga *Chlamydomonas reinhardtii*. Bioresour. Technol..

[191] Freudenberg R.A., Wittemeier L., Einhaus A., Baier T., Kruse O. (2022). Advanced Pathway Engineering for Phototrophic Putrescine Production. Plant Biotechnol. J..

[192] Fields F.J., Lejzerowicz F., Schroeder D., Ngoi S.M., Tran M., McDonald D., Jiang L., Chang J.T., Knight R., Mayfield S. (2020). Effects of the Microalgae *Chlamydomonas* on Gastrointestinal Health. J. Funct. Foods.

[193] Murbach T.S., Glávits R., Moghadam Maragheh N., Endres J.R., Hirka G., Goodman R.E., Lu G., Vértesi A., Béres E., Pasics Szakonyiné I. (2022). Evaluation of the Genotoxic Potential of Protoporphyrin IX and the Safety of a Protoporphyrin IX-Rich Algal Biomass. J. Appl. Toxicol..

[194] Grande T., Vornoli A., Lubrano V., Vizzarri F., Raffaelli A., Gabriele M., Novoa J., Sandoval C., Longo V., Echeverria M.C. (2023). *Chlamydomonas Agloeformis* from the Ecuadorian Highlands: Nutrients and Bioactive Compounds Profiling and In Vitro Antioxidant Activity. Foods.

[195] Bjørklund G., Gasmi A., Lenchyk L., Shanaida M., Zafar S., Mujawdiya P.K., Lysiuk R., Antonyak H., Noor S., Akram M. (2022). The Role of Astaxanthin as a Nutraceutical in Health and Age-Related Conditions. Molecules.

[196] Patel A.K., Tambat V.S., Chen C.W., Chauhan A.S., Kumar P., Vadrale A.P., Huang C.Y., Dong C.D., Singhania R.R. (2022). Recent Advancements in Astaxanthin Production from Microalgae: A Review. Bioresour. Technol..

[197] Perozeni F., Cazzaniga S., Baier T., Zanoni F., Zoccatelli G., Lauersen K.J., Wobbe L., Ballottari M. (2020). Turning a Green Alga Red: Engineering Astaxanthin Biosynthesis by Intragenic Pseudogene Revival in *Chlamydomonas reinhardtii*. Plant Biotechnol. J..

[198] Cazzaniga S., Perozeni F., Baier T., Ballottari M. (2022). Engineering Astaxanthin Accumulation Reduces Photoinhibition and Increases Biomass Productivity under High Light in *Chlamydomonas reinhardtii*. Biotechnol. Biofuels Bioprod..

[199] Tran Q.G., Cho K., Kim U., Yun J.H., Cho D.H., Heo J., Park S.B., Kim J.W., Lee Y.J., Ramanan R. (2019). Enhancement of Β-Carotene Production by Regulating the Autophagy-Carotenoid Biosynthesis Seesaw in *Chlamydomonas reinhardtii*. Bioresour. Technol..

[200] Del Mondo A., Smerilli A., Sané E., Sansone C., Brunet C. (2020). Challenging Microalgal Vitamins for Human Health. Microb. Cell Fact..

[201] Vidal-Meireles A., Neupert J., Zsigmond L., Rosado-Souza L., Kovács L., Nagy V., Galambos A., Fernie A.R., Bock R., Tóth S.Z. (2017). Regulation of Ascorbate Biosynthesis in Green Algae Has Evolved to Enable Rapid Stress-Induced Response via the VTC2 Gene Encoding GDP-l-Galactose Phosphorylase. New Phytol..

[202] Darwish R., Gedi M.A., Akepach P., Assaye H., Zaky A.S., Gray D.A. (2020). *Chlamydomonas reinhardtii* Is a Potential Food Supplement with the Capacity to Outperform *Chlorella* and *Spirulina*. Appl. Sci..

[203] Kamble P., Cheriyamundath S., Lopus M., Sirisha V.L. (2018). Chemical Characteristics, Antioxidant and Anticancer Potential of Sulfated Polysaccharides from *Chlamydomonas reinhardtii*. J. Appl. Phycol..

[204] Choudhary S., Save S.N., Vavilala S.L. (2018). Unravelling the Inhibitory Activity of *Chlamydomonas Reinhardtii* Sulfated Polysaccharides against α-Synuclein Fibrillation. Sci. Rep..

[205] Vishwakarma J., Vavilala S.L. (2019). Evaluating the Antibacterial and Antibiofilm Potential of Sulphated Polysaccharides Extracted from Green Algae *Chlamydomonas reinhardtii*. J. Appl. Microbiol..

[206] Arias C.A.D., de Oliveira C.F.M., Molino J.V.D., Ferreira-Camargo L.S., Matsudo M.C., Carvalho J.C.M.d. (2023). Production of Recombinant Biopharmaceuticals in *Chlamydomonas reinhardtii*. Int. J. Plant Biol..

[207] Torres-Tiji Y., Fields F.J., Yang Y., Heredia V., Horn S.J., Keremane S.R., Jin M.M., Mayfield S.P. (2022). Optimized Production of a Bioactive Human Recombinant Protein from the Microalgae *Chlamydomonas reinhardtii* Grown at High Density in a Fed-Batch Bioreactor. Algal Res..

[208] Kiefer A.M., Niemeyer J., Probst A., Erkel G., Schroda M. (2022). Production and Secretion of Functional SARS-CoV-2 Spike Protein in *Chlamydomonas reinhardtii*. Front. Plant Sci..

